# Protective effects of ten oligostilbenes from *Paeonia suffruticosa* seeds on interleukin-1β-induced rabbit osteoarthritis chondrocytes

**DOI:** 10.1186/s13065-019-0589-4

**Published:** 2019-05-23

**Authors:** Yu-Kun He, Xiao-tong Cen, Shuang-shuang Liu, Hua-ding Lu, Chun-nian He

**Affiliations:** 1 Department of Orthopedics, The Fifth Affiliated Hospital of Sun Yat-Sen University, Zhuhai, 519000 Guangdong China; 2 Guangzhou Institute of Biomedicine and Health, Chinese Academy of Sciences, Guangzhou, 510000 Guangdong China; 3 Institute of Medicinal Plant Development, Chinese Academy of Medical Science, Peking Union Medical College, Beijing, 100193 China

**Keywords:** *Paeonia suffruticosa*, Oligostilbene, Osteoarthritis, Chondrocyte

## Abstract

**Background:**

*Paeonia suffruticosa* is an important traditional Chinese herb used to treat osteoarthritis (OA) and oligostilbenes are the main active ingredient of the seeds of *P. suffruticosa*. The monomer *trans*-resveratrol of this species was demonstrated to have chondroprotective effects as a lead compound for the treatment of osteoarthritis, but it has not been applied due to its low efficacy.

**Methods:**

Oligostilbenes were isolated by chromatography and were identified by NMR and HPLC. A rabbit osteoarthritis chondrocyte model was induced by interleukin-1β and was treated with individual drugs to systematically evaluate their effects. Cell Counting Kit 8 was used to test their effects on cell viability, calculate EC_50_ and plot a dose–response curve.Their effects on apoptosis were analyzed by Annexin V and PI staining, and the expression of chondrocyte-specific genes COL2A1, MMP13 and SOX9 was evaluated by real-time PCR.

**Results:**

*Paeonia suffruticosa* seed extract could promote the cell viability of rabbit OA chondrocytes at low concentration and then ten oligostilbenes were isolated from it. *Trans*-oligostilbenes were better than their *cis*-forms, trimers and dimers were better than monomers for promoting the cell viability of rabbit osteoarthritis chondrocytes. None of the oligostilbenes was more effective than seed extract at the appropriate concentration; 1 μM oligostilbenes all showed various anti-apoptotic effects. *Trans*-gnetin H showed the best effect on proliferation and inhibition of MMP13 expression on OA chondrocytes, while *trans*-viniferin was most effective in promoting the expression of COL2A1 and SOX9.

**Conclusions:**

Ten oligostilbenes from *P. suffruticosa* seed all have certain protective effects on OA chondrocytes at low concentration. The *trans*-viniferin and some trimers have the potential to be further developed for the treatment of osteoarthritis because they were more effective than resveratrol and diacerein. The synergistic effect that may exist between oligostilbenes also warrants further research.

**Electronic supplementary material:**

The online version of this article (10.1186/s13065-019-0589-4) contains supplementary material, which is available to authorized users.

## Introduction

Osteoarthritis (OA), the most common chronic joint disease, is also one of the most common causes of disability. According to epidemiological studies, almost 15% of the population is affected by OA, and the incidence of OA is increasing by 100,000 people per year worldwide [1, 2]. However, there is currently no satisfactory treatment. Traditional drugs, such as nonsteroidal anti-inflammatory drugs (NSAIDS), can temporarily relieve symptoms, but the pathological process is not delayed. Surgery also cannot reverse the destruction of the articular cartilage, which eventually leads to the loss of joint function [3, 4]. Many studies have shown that the reduction of articular chondrocytes and the imbalance of extracellular matrix synthesis and degradation are the main pathological phenomena of OA [5]. Therefore, effectively delaying the degeneration of articular chondrocytes is the key to prevent or treat OA.

*Paeonia suffruticosa* is an important Chinese herbal medicine for the treatment of pain and inflammatory disease, including osteoarthritis [6]. Previously, scholars usually focused on its root extracts, such as paeonol or its analogues, as effective pharmaceutical ingredients, but other parts of the herb have not been fully studied [7, 8]. Recently, it was found that the seeds of *P. suffruticosa* are rich in oligostilbenes, including resveratrol and its dimers and trimers [9]. Natural oligostilbenes are one of the most important class of polyphenols, which are produced by the oligomerization or isomerization of resveratrol in nature. Resveratrol was discovered as phytoalexin and has a variety of pharmacological activities, such as anti-pathogenic activities toward microorganisms (bacteria, fungi and viruses) and anti-tumor and anti-inflammatory activities. Some studies have suggested that some of these oligomers have significantly stronger antioxidant, antitumor and anti-inflammatory activities than their monomer resveratrol and that the mechanisms are also different; these properties have inspired scholars to look for new drug candidates with high selectivity and few side effects [10–12]. However, there is still no effective total synthesis method because of their structural complexity. Additionally, most of these compounds are scarce in nature and are difficult to separate and structurally identify, which limits systematic research of their properties and activities. Therefore, finding high-content, high-activity oligostilbenes from plant resources and then artificially modifying them to improve their bioavailability and selectivity is the main way to study these compounds [13–15].

We found that *P. suffruticosa* seed extract has a protective effect on osteoarthritic chondrocytes at low concentrations, and then the main active ingredient oligostilbenes were identified from it. This study systematically evaluated the effects of these oligostilbenes on interleukin-1β(IL-1β)-induced OA chondrocyte models and determined their structure–activity relationships based on their effects on chondrocyte proliferation and extracellular matrix (ECM) secretion [16].

## Results

### Cell viability induced by seed extract of *Paeonia suffruticosa* on OA chondrocytes

**Fig. 1:**
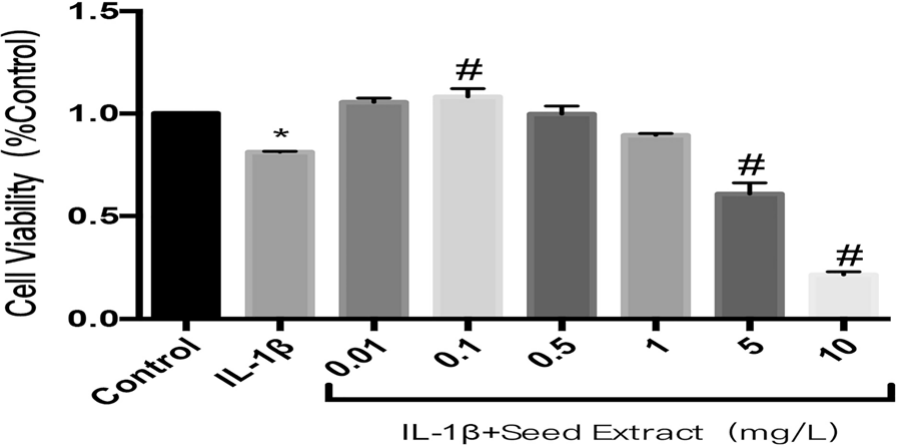
Effects of *Paeonia suffruticosa* seed extract on OA chondrocyte viability. Chondrocytes were treated with 10 ng/mL IL-1β and with different concentrations (0, 0.01, 0.1, 0.5, 1, 5 and 10 mg/L) of seed extract for 24 h. The cell viability was analyzed by CCK-8 (*compared with the control group, P < 0.05; #compared with the IL-1β group, P < 0.05)

The OA chondrocyte model was produced by 10 ng/mL IL-1β and untreated normal rabbit chondrocytes were used as a control group. Viewed under an inverted microscope, the rabbit chondrocytes in the control group were stretching better and grew faster in the form of typical paving-stones, while the cells in the IL-1β group were reduced in number, had a disordered arrangement and had a slender cell morphology. We also observed that the morphology and quantity of OA chondrocytes significantly recovered after treatment with low concentration of *P. suffruticosa* seed extract (shown in Additional file 1). Cell Counting Kit 8 (CCK-8) was used to further examined the effects of extract on rabbit OA chondrocyte viability. The result showed that the viability of OA chondrocytes was lower than that of normal chondrocytes. At the same time, we added different concentrations of extract to OA chondrocytes in each group and found that the extract at low concentrations (approximately 0.1 mg/L) promoted OA chondrocyte proliferation, while at higher concentrations (over 5 mg/L), the extract showed cytotoxicity (Fig. 1).

### Isolation and structure identification of ten oligostilbenes

**Fig. 2:**
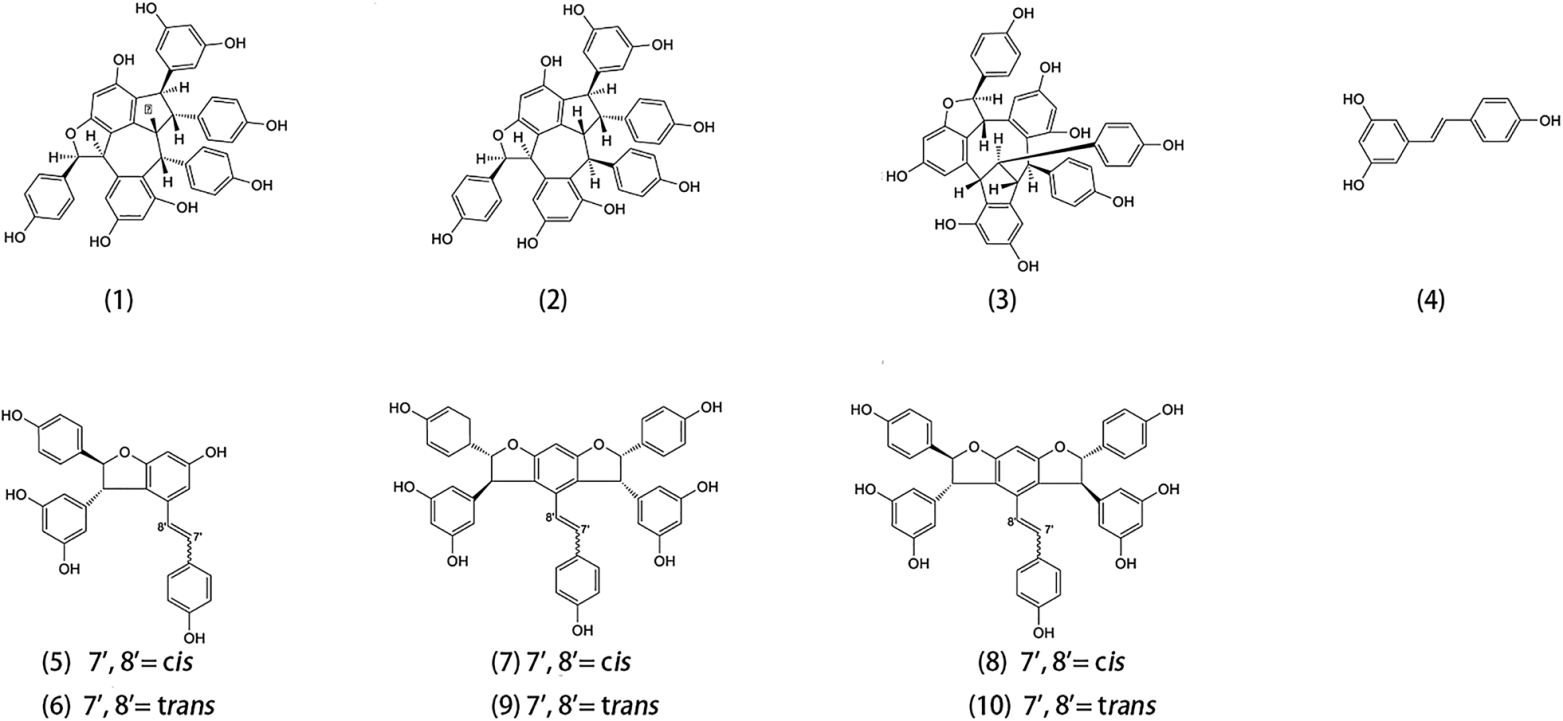
Chemical structures of ten oligostilbenes isolated fro*m the* seed shells of *Paeonia suffruticosa*. (1) suffruticosol A, (2) suffruticosol B, (3) suffruticosol C, (4) *trans*-resveratrol, (5) *cis*-ε-viniferin, (6) *trans*-ε-viniferin, (7) *cis*-suffruticosol D, (8) *cis*-gnetin H, (9) *trans*-suffruticosol D, and (10) *trans*-gnetin H. The number was based on their peak order of HPLC

Chromatography was applied to obtained ten oligostilbenes from *Paeonia suffruticosa* seed extract. Structural determinations of these ten oligostilbenes were elucidated by ^1^H- and ^13^C-NMR, including MS and UV techniques, and by comparison of those data with authentic compounds in published papers. These compounds were identified as suffruticosol A (1), suffruticosol B (2), suffruticosol C (3), *trans*-resveratrol (4), *cis*-ε-viniferin (5), *trans*-ε-viniferin (6), *cis*-suffruticosol D (7), *cis*-gnetin H (8), *trans*-suffruticosol D (9), and *trans*-gnetin H (10) and numbered based on their peak order of High Performance Liquid Chromatography (HPLC) (Fig. 2).

### Protective effects of ten oligostilbenes in rabbit osteoarthritis chondrocytes

**Fig. 3:**
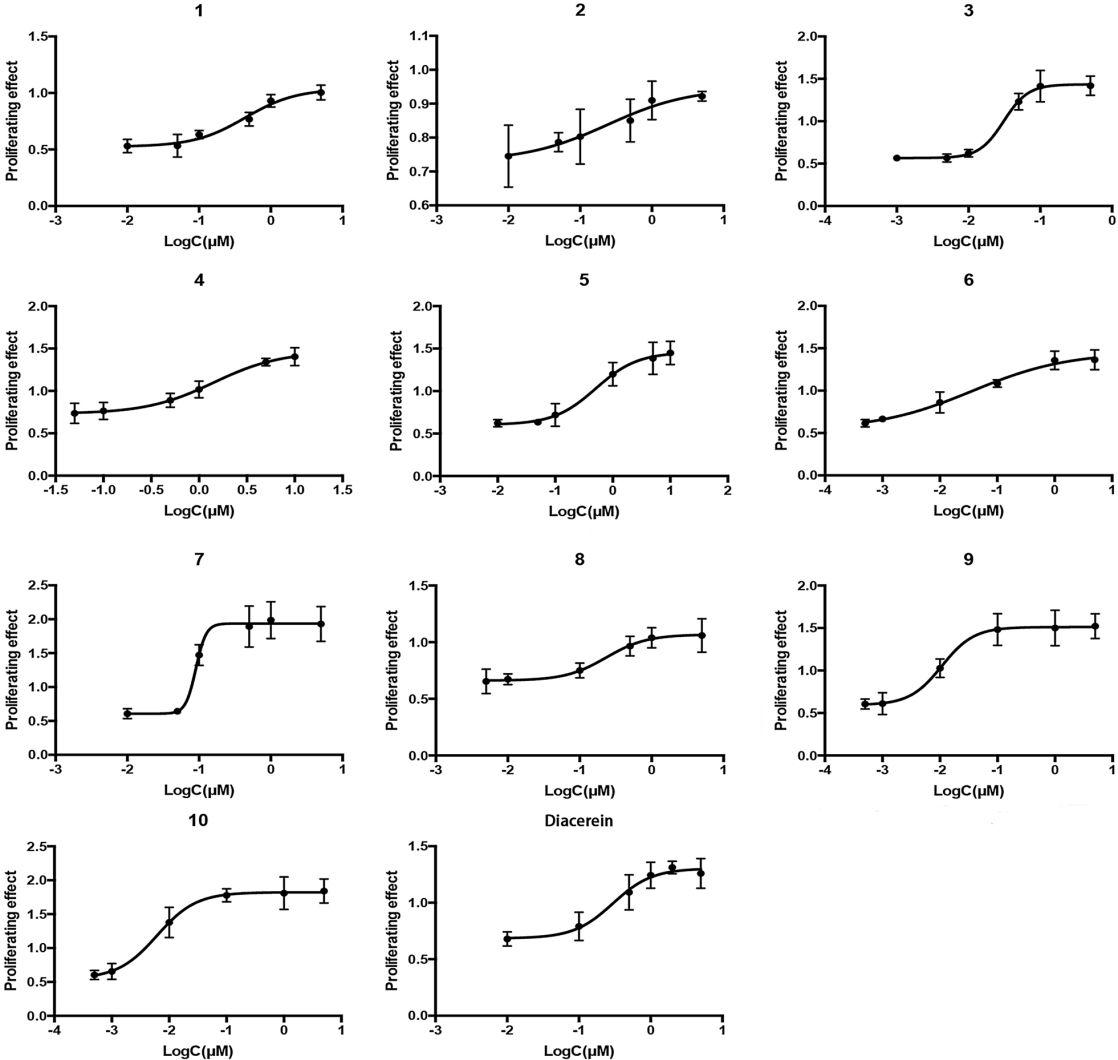
Dose–response curves of ten oligostilbenes and diacerein. Chondrocytes were treated with 10 ng/mL IL-1β and different concentrations of drug for 24 h. The chondrocytes’ absorbance values were measured by CCK-8. The abs*cis*sa represents the base-10 logarithm of drug concentration, and the ordinate represents the proliferative activity of drug, which was calculated according to the formula: (Experimental group − IL-1β group)/(untreated control group − IL-1β group). Data presented are the average of triplicates. Prism 6 was used to fit the curve and to calculate EC_50_

The proliferative activity of oligostilbenes of different concentrations on OA chondrocyte model was evaluated by CCK-8, and diacerein, known as an OA treatment drug and as an IL-1β inhibitor, was used as a positive control [17]. Their EC_50_ values were calculated by fitting their respective dose–effect relationship curves (Fig. 3). The results showed that all of them had antagonistic effects against the anti-proliferation effect of IL-1β at low concentrations. Even taking the 95% confidence intervals into account, the trimers and dimers were more effective than *trans*-resveratrol, and the trans isomers were more effective than the corresponding cis isomer. *Trans*-gnetin H had the best proliferative activities with an EC_50_ that was significantly lower than that of diacerein (Table 1).

**Table 1:** EC_50_ values (µM) of ten oligostilbenes antagonize the anti-proliferative effect of IL-1β on rabbit chondrocytes

No.	Name	DP	EC_50_ (μM)	95% confidence intervals (μM)
1	suffruticosol A	3	0.439	0.221 to 0.872
2	suffruticosol B	3	0.242	0.013 to 4.439
3	suffruticosol C	3	0.031	0.019 to 0.050
4	*trans*-resveratrol	1	1.416	0.520 to 3.857
5	*cis*‐*ε*-viniferin	2	0.506	0.209 to 1.228
6	*trans*‐*ε*-viniferin	2	0.037	0.009 to 0.152
7	*cis*-suffruticosol D	3	0.090	0.068 to 0.121
8	*cis*-gnetin H	3	0.233	0.086 to 0.627
9	*trans*-suffruticosol D	3	0.011	0.006 to 0.019
10	*trans*-gnetin H	3	0.006	0.002 to 0.019
Control	Diacerein	–	0.272	0.136 to 0.654

DP, degree of polymerization; EC_50_, concentration for 50% of maximal effect

### Changes in cell viability induced by 0.1 mg/L seed extract of *Paeonia suffruticosa* and ten oligostilbenes

**Fig. 4:**
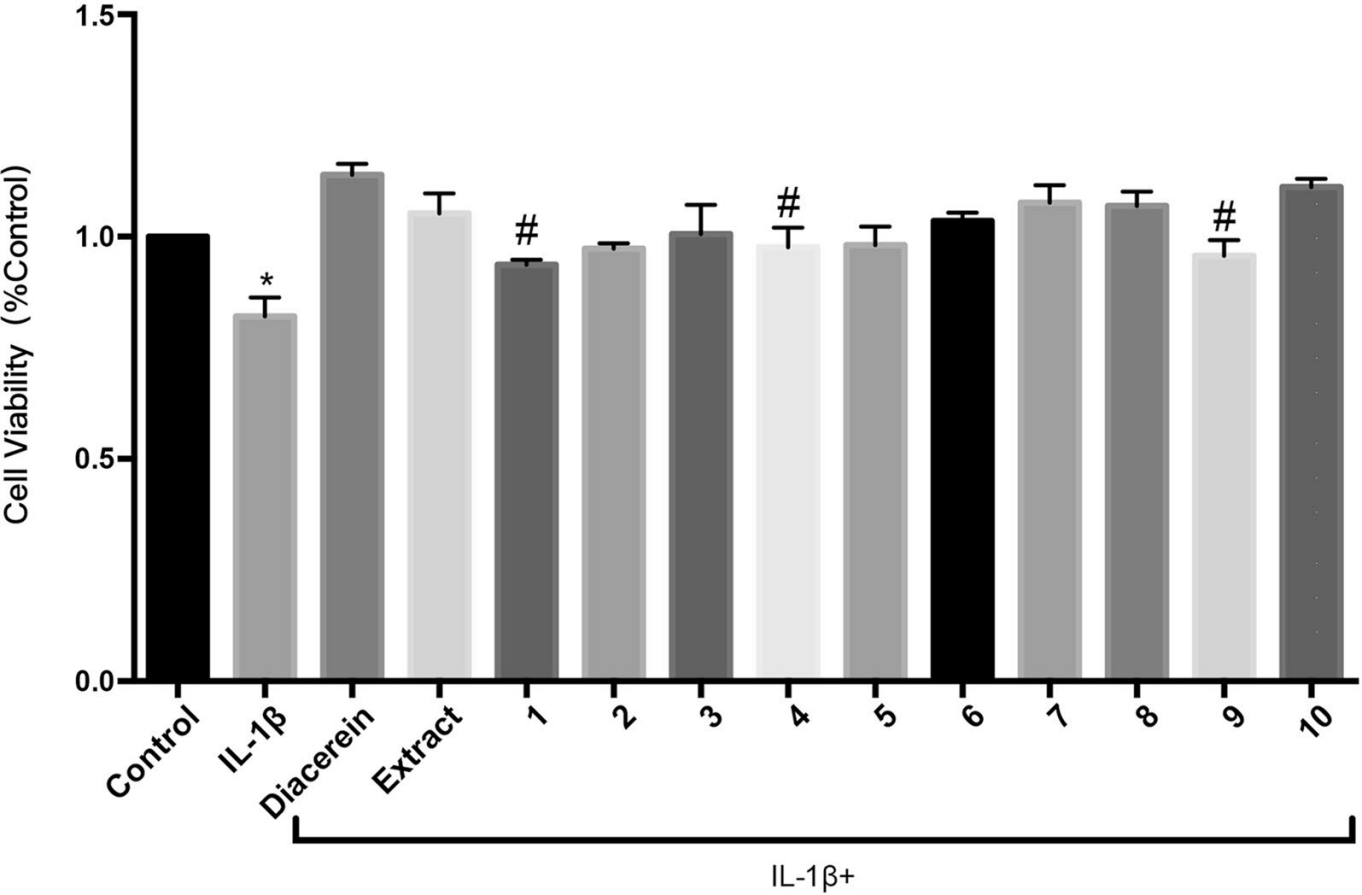
Effects of *Paeonia suffruticosa* seed extract and ten oligostilbenes on OA chondrocyte viability. Cells were treated with 10 ng/mL IL-1β and 0.1 mg/L of a different drug for 24 h and untreated chondrocytes were used as control group. The cell viability was analyzed by CCK-8 (*comparing with control group, P < 0.05; #comparing with IL-1β + extract treated group, P < 0.05). (1) suffruticosol A, (2) suffruticosol B, (3) suffruticosol C, (4) *trans*-resveratrol, (5) *cis*-ε-viniferin, (6) *trans*-ε-viniferin, (7) *cis*-suffruticosol D, (8) *cis*-gnetin H, (9) *trans*-suffruticosol D, and (10) *trans*-gnetin H

We further tested the effects of the extract and ten oligostilbenes on rabbit OA chondrocytes with diacerein as a positive control (shown in Additional file 2). The results showed that all drugs could promote the viability of OA chondrocytes at 0.1 mg/L (P < 0.05), but there was no significant difference between the effects of extract and diacerein. Compared with the extract, the proliferative effect of each oligostilbene was similar or lower, indicating that there might be synergistic effects among these compounds, which could improve the overall protective effect of their mixture (Fig. 4).

### Anti-apoptotic effects of ten oligostilbenes in rabbit osteoarthritis chondrocytes

**Fig. 5:**
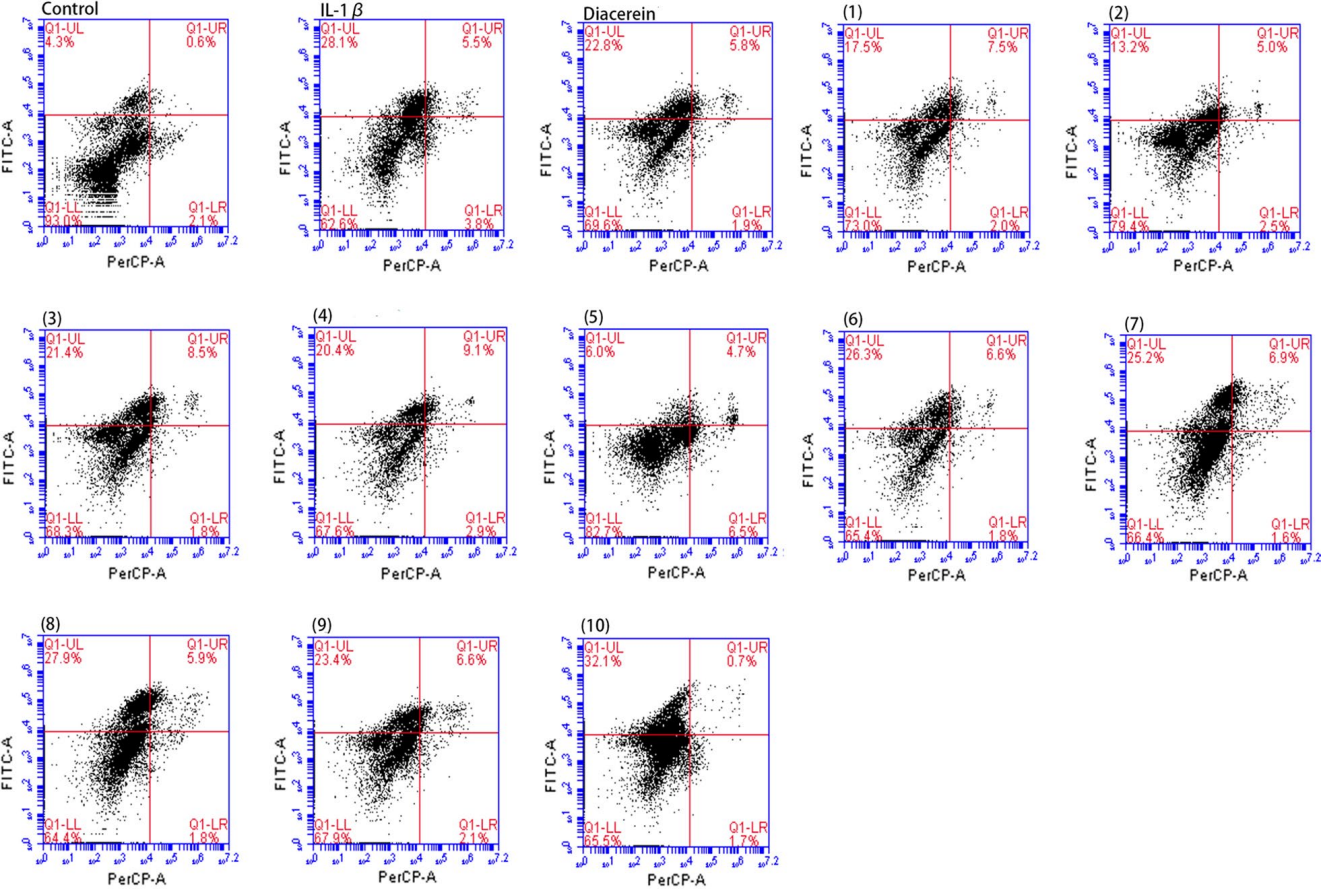
Effects of ten oligostilbenes on apoptosis of OA chondrocytes. Group of experiments: untreated control group, IL-1β group, the following groups were treated with 10 ng/mL IL-1β in addition to 1 μM the listed drug: diacerein, (1) suffruticosol A, (2) suffruticosol B, (3) suffruticosol C, (4) *trans*-resveratrol, (5) *cis*-ε-viniferin, (6) *trans*-ε-viniferin, (7) *cis*-suffruticosol D, (8) *cis*-gnetin H, (9) *trans*-suffruticosol D, and (10) *trans*-gnetin H

For further analyzing the mechanism of each drug in protecting OA chondrocytes in vitro, the apoptosis rate was analyzed by flow cytometry. Annexin V-FITC was an indicator of early cell apoptosis and was detected by the FITC-A channel, while PI was an indicator of late cell apoptosis and was detected by the PerCP-A channel. Untreated normal rabbit chondrocytes were used as a control group. After inducing the rabbit chondrocytes with 10 ng/mL IL-1β, they were treated with 1 μM drug separately for 24 h. The result showed the proportion of normal cells decreased from 93 to 62.6%, while the cells with early apoptosis increased from 4.3 to 28.1% under the action of IL-1β. The apoptosis rates declined to various degrees after the addition of each drug. The effect of *cis*-viniferin, suffruticosol A, and suffruticosol B were relatively significant, with the proportion of normal cells rising to 82.7%, 73%, and 79.4%, respectively (Fig. 5).

### Changes in chondrocyte-specific gene expression induced by ten oligostilbenes

**Fig. 6:**
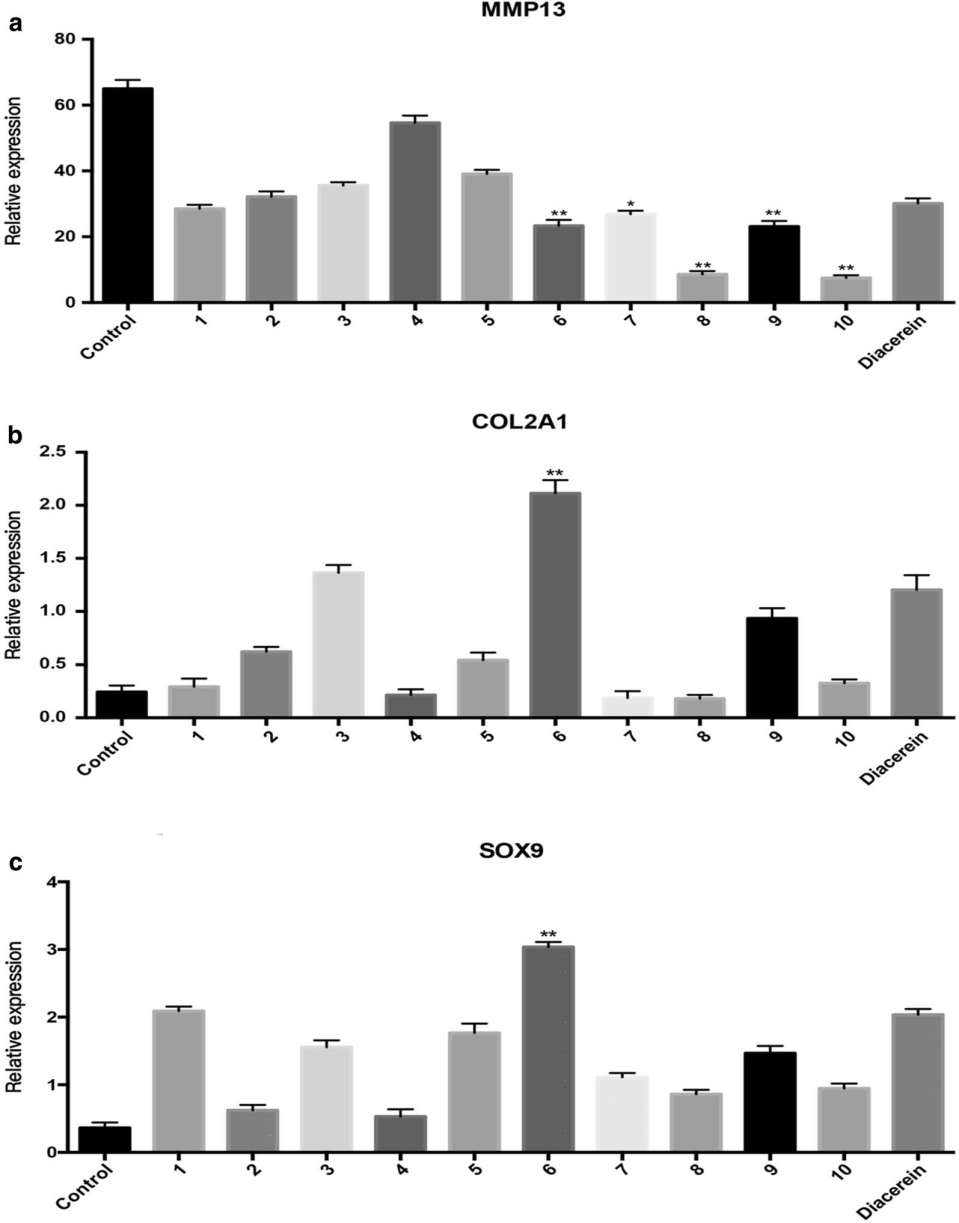
Effects of ten oligostilbenes on the gene expression of MMP13 (**a**), COL2A1 (**b**), SOX9 (**c**) of OA chondrocytes. Cells were treated with 10 ng/mL IL-1β and 1 μM test compound for 24 h. Diacerein (1 µM) was used as a positive control, and cells treated with IL-1β only served as a negative control. The gene expression was assayed by Q-PCR (compared with the diacerein group, *P < 0.05, **P < 0.01). (1) suffruticosol A, (2) suffruticosol B, (3) suffruticosol C, (4) *trans*-resveratrol, (5) *cis*-ε-viniferin, (6) *trans*-ε-viniferin, (7) *cis*-suffruticosol D, (8) *cis*-gnetin H, (9) *trans*-suffruticosol D, and (10) *trans*-gnetin H

Under the action of IL-1β, rabbit normal chondrocytes showed increased expression of MMP13, decreased expression of collagen II and Sox9 and other characteristics of OA chondrocytes. For determining the effect of the ten oligostilbenes on phenotype and ECM secretion of OA chondrocytes, we evaluated the expression of chondrocyte-specific genes MMP13, COL2A1, and SOX9 in each group. Compared with the negative control group, the expression of COL2A1 and SOX9 in chondrocytes that were treated with oligostilbenes were all increased, and the expression of MMP13 was decreased (P < 0.05), which indicated an inhibition of ECM degradation and the phenotypic stability of chondrocytes. Compared with the positive control group, trans-viniferin, cis/trans-suffruticosol D, and cis/trans-gnetin H reduced the expression of MMP13, and trans-viniferin showed the best effect to promote the expression of COL2A1 and SOX9, which means that trans-viniferin might be a better protector of chondrocytes in OA (Fig. 6).

## Discussion

*Paeonia suffruticosa* are cultivated in a large area, but their seeds have not been effectively used to date [18]. Our results indicated that *Paeonia suffruticosa* seed extract has a protective effect on OA chondrocytes at low concentrations. The next question is exactly which component is working. Previous studies suggested that resveratrol and its oligomers in the seeds of *P. suffruticosa* are mainly found in their seed coat with a content of more than 16.7%, which is significantly higher than other natural plants. In addition, these oligostilbenes can be fully enriched after extraction (total scorpion content > 75%), which is beneficial to their research and utilization [19].

As one of the most promising lead compounds for OA treatment, the protective effect of resveratrol on chondrocytes has been widely demonstrated in vivo and in vitro, but the low bioavailability due to its rapid metabolism reduced its pharmacological effects [20]. Studies showed resveratrol derivatives might overcome these shortcomings. For example, gnetin C, a resveratrol dimer, improved bioavailability compared to resveratrol [21]. This study systematically evaluated the effects of 10 oligostilbenes isolated from *P. suffruticosa* seeds on IL-1β-induced rabbit OA chondrocyte models and found that some of the dimers and trimers had better ability to protect OA chondrocytes compared with resveratrol, and their bioavailability can be further studied.

The analysis of their structure–activity relationship revealed that the *trans*-isomerism is important for their proliferative activity to rabbit OA chondrocytes. Our results suggested that the EC_50_ of three cis isomers were significantly higher than their corresponding trans isomer, which agreed with our current knowledge that the trans isomer of natural compounds is a more active form than cis because of its lower steric hindrance [22]. The activity of oligostilbenes to promote chondrocyte proliferation often increased with the degree of polymerization, which was consistent with their structure–antioxidant activity [23, 24]. Additionally, both of *cis*-suffruticosol D and *cis*-gnetin H are trimers that differ only in their three-dimensional structures but had significant differences in their efficacies, which indicated that the effect of three-dimensional structure is also worth paying attention.

Studies have shown that there is a synergistic effect between some oligostilbenes during the anti-inflammatory process; therefore, we further explored whether this synergistic effect exists among these 10 compounds [25]. According to previous results, 0.1 mg/L of the extract or oligostilbenes were selected for detection, but no significant change was found between the protective effect of extract and oligostilbenes. However, resveratrol, suffruticosol A and *trans*-suffruticosol D were slightly less effective than extracts; therefore, there might still be some synergy in the mixture. However, it was not obvious because their content in the extract was too low, or the ingredients were too complicated. Therefore, further specific research is necessary.

Chondrocyte apoptosis is the initial step in the pathogenesis of OA. In OA cartilage, we could observe an increase in apoptotic chondrocytes and a change in the expression of apoptosis-related genes [26]. The OA chondrocyte model induced by IL-1β in this study also showed the same characteristics. It was found that oligostilbenes could reduce apoptotic chondrocytes, but most of them had no obvious advantage compared with diacerein, and their anti-apoptotic ability had no significant correlation with their proliferative activities in chondrocytes. Therefore, the anti-apoptosis ability of oligostilbenes in OA chondrocytes may not be the main reason for their efficiency of protecting chondrocytes.

Chondrocytes are quiescent cells that rarely regenerate in vivo, and the ECM is all produced by them without nerves or vessels. As a result, the survival of chondrocytes and their phenotypic stability and anabolic/catabolic balance activity are important for the maintenance of articular cartilage [27]. We further analyzed the changes of chondrocyte gene expression under the action of oligostilbenes, including matrix metalloproteinase 13(MMP13), an enzyme that is involved in the degradation of extracellular matrix (ECM) in OA [28]; COL2A1, the gene that provides type II collagen, which is the main protein in ECM of chondrocytes [29]; SOX9, an important factor in promoting cartilage differentiation, and its expression is decreased in OA patients [30]. We found that they all have the ability to inhibit the ECM degradation of chondrocytes and promote the expression of chondrocyte-specific genes. Furthermore, the different MMP13 expression efficiencies of the oligostilbenes in chondrocytes were consistent with their efficiencies of promoting chondrocyte viability. Therefore, we could further speculate that oligostilbenes mainly protect chondrocytes through inhibiting ECM degradation. Compared with diacerein, *trans*-viniferin was better at promoting the expression of COL2A1 and SOX9 in chondrocytes, which means that *trans*-viniferin has the potential to be a more effective OA drug.

There were also some unclear places in our results that needed further explanation. For example, the ability of oligostilbenes to degrade ECM was not consistent with their ability to promote the expression of Collagen II in chondrocytes. However, the impact of oligostilbenes on the survival and maintenance of chondrocytes is very complicated, and further research about its mechanism is still necessary.

## Conclusions

Our results indicated that ten oligostilbenes from *P. suffruticosa* seed all have certain protective effects on OA chondrocytes at low concentration. *Trans*-viniferin and some trimers were better at promoting cell viability, reducing apoptosis, or maintaining the phenotypic stability of OA chondrocytes than resveratrol and diacerein; thus, they deserved further research to overcome the shortcoming of resveratrol in clinical using for the treatment of osteoarthritis. The combined usage of oligostilbenes may be a way to improve their efficacy.

## Materials and methods

The study did not involve animal or human experiments; thus, no ethical approvals are required.

### Materials

Rabbit primary chondrocytes (iCell Bioscience Inc., Shanghai, China); Fetal Bovine Serum (Gibco, 10099141); Trypsin 0.25% EDTA (Gibco, 25200114); DMEM High Glucose (HyClone, SH30022.01); Penicillin–Streptomycin Solution (HyClone, SV30010); DPBS (Gibco, C14190500BT); Recombinant Human IL-1β (Invitrogen, USA); Cell Counting Kit-8 (Keygen, Nanjing, China); Annexin V-FITC/PI Apoptosis Detection Kit (Keygen, Nanjing, China); Diacerein (Aladdin, Shanghai, China); dimethyl sulfoxide (Sigma-Aldrich, Germany); RNase-free water (Tiangen, Beijing, China); Revertra Ace (TOYOBO, Japan); Recombinant Rnasin Ribonuclcase Inhibitor (Promega, USA); Advanced SYBR Green Supermix (Bio-Rad, USA); Oligo (dT) 18 Primer (TaKara, Japan); TRI Reagent (MRC, USA); HPLC-grade MeOH (Fisher Scientific, NJ, USA). Analytical-grade solvents for extraction and chromatography (Beijing Beihua Fine Chemicals Company, Beijing, China); ODS-A C18 reversed-phase silica gel (50 μm, YMC).

### Plant material

The seed shells of *Paeonia suffruticosa* were obtained from Heze, Shandong province, P. R. China and were identified by Dr. Chun-nian He. The voucher specimen (2012001) has been deposited in the Seed Resource Bank of the Institute of Medicinal Plant Development, Chinese Academy of Medical Sciences and Peking Union Medical College, Beijing, P. R. China.

### Extraction and isolation

The dried seed shells (1.2 kg) were pulverized and extracted with 70% ethyl alcohol (EtOH,9 L) by soaking at room temperature for 24 h twice. The combined EtOH extract was concentrated under reduced pressure at 60 °C to afford a dark-brown residue (245 g). A portion of the EtOH extract of the seed shells (120 g) that was dissolved in MeOH–H_2_O (60: 40, v/v) was chromatographed through an ODS-A C18 reversed-phase silica gel column (5.0 cm i.d. × 50 cm) eluted with MeOH-H_2_O (20: 80 → 80: 20, v/v) gradient to afford fractions A-D. Fraction B (40 g) was rechromatographed through a YMC-pack ODS-A column (250 mm × 20 mm, 5 μm) on pre-HPLC (BUCHI Reveleris PREP) eluted with MeOH-H_2_O (40: 60, v/v) to obtain suffruticosol A (**1**, 2.0 g), suffruticosol B (**2**, 5.0 g), and suffruticosol C (**3**, 55 mg), respectively. Fraction C (20 g) was rechromatographed through a YMC-pack ODS-A column (250 mm × 20 mm, 5 μm) on pre-HPLC (BUCHI Reveleris PREP) eluted with MeOH-H_2_O (45: 55, v/v) to obtain *trans*-resveratrol (**4**800 mg), *cis*-ε-viniferin (**5**200 mg), and *trans*-ε-viniferin (**6**, 1.2 g), respectively. Fraction D (38 g) was rechromatographed through a YMC-pack ODS-A column (250 mm × 20 mm, 5 μm) on pre-HPLC (BUCHI Reveleris PREP) eluted with MeOH-H_2_O (52: 48, v/v) to obtain *cis*-suffruticosol D (**7**, 55 mg), *cis*-gnetin H (**8**, 45 mg), *trans*-suffruticosol D (**9**, 1.5 g), and *trans*-gnetin H (**10**, 5.2 g), respectively (flowchart was shown in Additional file 3).

Confirmation of the ten compound was made by comparison with the reference substance isolated previously by the author [9]. ^1^H- and ^13^C-NMR spectra were measured on a Bruker Avance DRX-500 spectrometer (^1^H at 500 MHz and ^13^C at 125 MHz) in MeOH-*d*4. Chemical shifts are given in δ values (ppm) relative to tetramethylsilane (TMS) as an internal standard (shown in Additional file 4). UV spectra were measured on a Shimadzu UV-2550 UV–visible spectrophotometer. ESI–MS spectra were recorded on an ABI 3200 Qtrap spectrometer.

#### Suffruticosol A (1)

UV λ_max_ nm: 230, 285 (MeOH). ESI–MS *m/z*: 679 (M^+^ - H). ^1^H-NMR (500 MHz, CD_3_OD) δ (ppm): 7.10 (2H, d, *J *= 8.5 Hz, H-2″, 6″), 6.95 (2H, d, *J *= 8.5 Hz, H-2, 6), 6.69 (2H, d, *J *= 8.5 Hz, H-3″, 5″), 6.48 (2H, d, *J *= 8.5 Hz, H-2′, 6′), 6.36 (2H, d, *J *= 8.5 Hz, H-3, 5), 6.24 (1H, d, *J *= 2.5 Hz, H-12″), 6.19 (1H, d, *J *= 1.0 Hz, H-12′), 6.11 (2H, d, *J *= 8.5 Hz, H-3′, 5′), 6.05 (1H, t, *J *= 2.5 Hz, H-12), 5.98 (2H, d, *J *= 2.5 Hz, H-10, 14), 5.93 (1H, d, *J *= 2.5 Hz, H-14″), 5.68 (1H, d, *J *= 12.0 Hz, H-7″), 5.42 (1H, d, *J *= 3.5 Hz, H-7′), 4.74 (1H, s, H-8), 4.34 (1H, d, *J *= 12.0 Hz, H-8″), 3.93 (1H, m, H-8′), 3.68 (1H, d, *J *= 8.0 Hz, H-8). ^13^C-NMR (125 MHz, CD_3_OD) δ (ppm): 160.7 (C-11′), 159.8 (C-11, 13), 159.4 (C-4″), 157.2 (C-13″), 157.0 (C-4), 155.6 (C-13′), 155.4 (C-11″), 155.0 (C-4′), 148.9 (C-9), 145.1 (C-9′), 142.3 (C-9″), 136.0 (C-1), 134.4 (C-1′), 131.3 (C-1″), 131.2 (C-2′, 6′), 131.0 (C-2, 2″, 6, 6″), 127.4 (C-10″), 123.5 (C-14′), 117.7 (C-10′), 116.7 (C-3″, 5″), 115.9 (C-3, 5), 114.7 (C-3′, 5′), 107.3 (C-10, 14), 106.4 (C-14″), 102.4 (C-12″), 101.9 (C-12), 96.7 (C-12′), 92.0 (C-7″), 61.5 (C-7), 55.0 (C-8), 49.5 (C-8″), 49.1 (C-8′), 40.2 (C-7′).

#### Suffruticosol B (2)

UV λ_max_ nm: 230, 284 (MeOH). ESI–MS *m/z*: 679 (M^+^ - H). ^1^H-NMR (500 MHz, CD_3_OD) δ (ppm): 7.58 (2H, d, *J *= 8.5 Hz, H-2″, 6″), 6.95 (2H, brd, H-2′, 6′), 6.91 (2H, d, *J *= 8.5 Hz, H-3″, 5″), 6.50 (2H, d, *J *= 8.5 Hz, H-3′, 5′), 6.28 (2H, d, *J *= 8.5 Hz, H-3, 5), 6.26 (2H, d, *J *= 8.5 Hz, H-2, 6), 6.22 (1H, d, *J *= 2.0 Hz, H-10, 14), 6.19 (1H, s, H-12′), 6.17 (2H, d, *J *= 2.0 Hz, H-12″), 6.16 (1H, t, *J *= 2.0 Hz, H-12), 5.94 (2H, d, *J *= 2.5 Hz, H-14″), 5.86 (1H, d, *J *= 11.5 Hz, H-7″), 5.08 (1H, d, *J *= 11.5 Hz, H-8″), 4.22 (1H, d, *J *= 11.5 Hz, H-7′), 4.11 (1H, m, H-8′), 4.09 (1H, s, H-8), 3.81 (1H, d, *J *= 6.0 Hz, H-7). ^13^C-NMR (125 MHz, CD_3_OD) δ (ppm): 160.7 (C-11′), 159.9 (C-11, 13), 159.6 (C-4″), 158.9 (C-13″), 157.7 (C-11″), 156.6 (C-4′), 156.5 (C-4), 156.2 (C-13′), 148.0 (C-9, 9′), 142.9 (C-9″), 136.0 (C-1), 134.3 (C-1′), 133.6 (C-2′, 6′), 131.4 (C-1″), 131.0 (C-2″, 6″), 129.9 (C-2, 6), 124.1 (C-14′), 123.4 (C-10″), 119.0 (C-10′), 117.0 (C-3″, 5″), 115.6　(C-3, 5), 115.2 (C-3′, 5′), 107.8 (C-10, 14), 105.4 (C-12″), 104.2 (C-14″), 101.9 (C-12), 96.7 (C-12′), 91.6 (C-7″), 63.6 (C-7), 57.4 (C-8), 49.3 (C-8″), 48.3 (C-8′), 47.0 (C-7′).

#### Suffruticosol C (3)

UV λ_max_ nm: 230, 285 (MeOH). ESI–MS *m/z*: 679 (M^+^ - H). ^1^H-NMR (500 MHz, CD_3_OD) δ (ppm): 7.23 (2H, d, *J *= 8.4 Hz, H-2′, 6′), 7.12 (2H, d, *J *= 8.4 Hz, H-2″, 6″), 6.95 (2H, d, *J *= 9.0 Hz, H-2, 6), 6.72 (2H, d, *J *= 7.8 Hz, H-3′, 5′, 3″, 5″), 6.54 (2H, d, *J *= 8.4 Hz, H-3, 5), 6.41 (1H, d, *J *= 2.5 Hz, H-10″), 6.27 (1H, d, *J *= 2.5 Hz, H-12″), 6.03 (3H, s, H-7″, 12, 12′), 5.89 (2H, d, *J *= 2.5 Hz, H-10, 10′), 5.10 (1H, s, H-7), 4.29 (1H, d, *J *= 9.6 Hz, H-8′), 4.26 (1H, s, H-8″), 4.17 (1H, d, *J *= 11.4 Hz, H-8), 3.01 (1H, dd, *J *= 10.2, 11.4 Hz, H-7′).

#### Resveratrol (4)

UV λ_max_ nm: 219,230, 285 (MeOH). ESI–MS *m/z*: 227 (M^+^ - H). ^1^H-NMR (500 MHz, CD_3_OD) δ (ppm): 7.04 (2H, d, *J *= 8.5 Hz, H-2, 6), 6.61 (2H, d, *J *= 8.5 H-3, 5), 6.36 (1H, d, *J *= 12.0 Hz, H-7), 6.26 (1H, d, *J *= 12.0 Hz, H-8), 6.18 (2H, d, *J *= 2.5 H-10, 14), 6.10 (1H, t, *J *= 2.5 Hz, H-12). ^13^C-NMR (125 MHz, CD_3_OD) δ (ppm): 159.8 (C-11, 13), 158.2 (C-4), 141.5 (C-9), 131.3 (C-7), 130.6 (C-2, 6), 130.3 (C-1), 129.6 (C-8), 116.4 (C-3, 5), 108.5 (C-10, 14), 102.9 (C-12).

#### *cis*-*ε*-Viniferin (5)

UV λ_max_ nm: 230, 280 (MeOH). ESI–MS *m/z*: 453 (M^+^ - H). ^1^H-NMR (500 MHz, CD_3_OD) δ (ppm): 6.94 (2H, d, *J *= 8.4 Hz, H-2, 6), 6.93 (2H, d, *J *= 8.4 Hz, H-2′, 6′), 6.71 (2H, d, *J *= 8.4 Hz, H-3, 5), 6.59 (2H, d, *J *= 8.4 H-3′, 5′), 6.24 (1H, d, *J *= 2.4 H-14′), 6.22 (1H, d, *J *= 2.4 Hz, H-12′), 6.20 (1H, d, *J *= 12.0 H-7′), 6.09 (1H, t, *J *= 2.4 Hz, H-12), 6.03 (1H, d, *J *= 12.0 Hz, H-8′), 5.93 (2H, d, *J *= 2.4 Hz, H-10, 14), 5.18 (1H, d, *J *= 6.0 Hz, H-7), 3.78 (1H, d, *J *= 6.0 Hz, H-8). ^13^C-NMR (125 MHz, CD_3_OD) δ (ppm): 163.2 (C-11′), 160.1 (C-11, 13), 159.6 (C-13′), 159.0 (C-4′), 158.3 (C-4), 147.7 (C-9), 137.9 (C-9′), 134.3 (C-1), 131.8 (C-7′), 131.2 (C-2′, 6′), 130.6 (C-1′), 128.8 (C-2, 6), 127.2 (C-8′), 120.8 (C-10′), 116.4 (C-3′, 5′), 116.6 (C-3, 5), 109.4 (C-14′), 107.9 (C-10, 14), 102.3 (C-12), 97.2 (C-12′), 95.4 (C-7), 58.2 (C-8).

#### *trans*-*ε*-Viniferin (6)

UV λ_max_ nm: 230, 330 (MeOH). ESI–MS *m/z*: 453 (M^+^ - H). ^1^H-NMR (500 MHz, CD_3_OD) δ (ppm): 7.14 (2H, d, *J *= 9.0 Hz, H-2, 6), 7.03 (2H, d, *J *= 9.0 Hz, H-2′, 6′), 6.81 (1H, d, *J *= 16.0 Hz, H-7′), 6.76 (2H, d, *J *= 9.0 Hz, H-3, 5), 6.65 (2H, d, *J *= 9.0 H-3′, 5′), 6.63 (1H, d, *J *= 2.0 H-14′), 6.56 (1H, d, *J *= 16.0 Hz, H-8′), 6.25 (1H, d, *J *= 2.0 Hz, H-12′), 6.18 (1H, t, *J *= 2.0 Hz, H-12), 6.16 (2H, d, *J *= 2.0 Hz, H-10, 14), 5.36 (1H, d, *J *= 6.5 Hz, H-7), 4.35 (1H, d, *J *= 6.5 Hz, H-8). ^13^C-NMR (125 MHz, CD_3_OD) δ (ppm): 163.2 (C-11′), 160.5 (C-11, 13), 160.2 (C-13′), 159.0 (C-4′), 158.9 (C-4), 147.9 (C-9), 137.4 (C-9′), 134.4 (C-1), 130.9 (C-7′), 130.8 (C-1′), 129.3 (C-2′, 6′), 128.7 (C-2, 6), 124.2 (C-8′), 120.6 (C-10′), 116.9 (C-3′, 5′), 116.8 (C-3, 5), 108.0 (C-10, 14), 104.9 (C-14′), 102.7 (C-12), 97.4 (C-12′), 95.3 (C-7), 58.8 (C-8).

#### *cis*-Suffruticosol D (7)

UV λ_max_ nm: 203, 230 (sh), 282, 328 (MeOH). ESI–MS *m/z*: 679 (M^+^–H). ^1^H-NMR (500 MHz, CD_3_OD) δ (ppm): 7.17 (2H, d, *J *= 8.5 Hz, H-2, 6), 6.95 (2H, d, *J* = 8.5 Hz, H-2″, 6″), 6.78 (2H, d, *J *= 8.5 Hz, H-3, 5), 6.75 (2H, d, *J* = 8.5 Hz, H-2′, 6′), 6.55 (2H, d, *J* = 8.5 Hz, H-3″, 5″), 6.54 (2H, d, *J *= 8.5 Hz, H-3′, 5′), 6.42 (H, d, *J *= 12.0 Hz, H-7′), 6.46 (H, s, H-12′), 6.36 (H, d, *J* = 12.0 Hz, H-8′), 6.19 (H, t, *J *= 2.0 Hz, H-12), 6.17 (2H, d, *J *= 2.0 Hz, H-10,14), 5.94 (H, t, *J* = 2.0 Hz, H-12″), 5.82 (H, d, *J* = 8.0 Hz, H-7″), 5.78 (2H, d, *J* = 2.0 Hz, H-10″,14″), 5.43 (H, d, *J *= 6.0 Hz, H-7), 4.50 (H, d, *J* = 8.0 Hz, H-8″), 4.39 (H, d, *J *= 6.0 Hz, H-8). ^13^C-NMR (125 MHz, CD_3_OD) δ (ppm): 162.6 (C-11′13′), 159.9 (C-11, 13), 159.0 (C-11″,13″), 158.9 (C-4), 158.4 (C-4′), 157.9 (C-4″), 148.3 (C-9), 143.9 (C- 9″), 135.1 (C-7′), 134.7 (C-1), 133.9(C-9′), 131.1 (C-1′), 130.1 (C-1″), 129.8(C-2″,6″), 129.5 (C-2′, 6′), 128.7 (C-2, 6), 125.0(C-8′), 122.4(C-14′), 121.6 (C-10′), 116.7 (C-3, 5), 116.4(C-3″,5″), 115.8 (C-3′, 5′), 109.8(C-10″,14″), 107.9 (C-10, 14), 102.3 (C-12), 102.0 (C-12″), 95.7 (C-7), 92.3 (C-12′), 91.6 (C-7″), 59.3 (C-8), 55.3(C-8″).

#### *cis*-gnetin H (8)

UV λ_max_ nm: 203, 230 (sh), 282 (MeOH). ESI–MS *m/z*: 679 (M^+^ –H). ^1^H-NMR (500 MHz, CD_3_OD) δ (ppm): 6.98 (4H, d, *J* = 8.5 Hz, H-2,6,2″6″), 6.77 (2H, d, *J *= 8.5 Hz, H-2′, 6′), 6.69(4H, d, *J *= 8.5 Hz, H-3,5,3″, 5″), 6.50 (2H, d, *J *= 8.5 Hz, H-3′, 5′), 6.35 (H, s, H-12′), 6.05 (2H, t, *J *= 2.0 Hz, H-12,12″), 5.95 (2H, d, *J *= 2.0 Hz, H-10,14), 5.92 (H, d, *J *= 12.0 Hz, H-7′), 5.78(2H, d, *J* = 2.0 Hz, H-10″,14″), 5.74 (H, d, *J* = 12.0 Hz, H-8′), 5.24 (2H, d, *J* = 5.0 Hz, H-7,7″), 3.86 (2H, d, *J* = 5.0 Hz, H-8,8″). ^13^C-NMR (125 MHz, CD_3_OD) δ(ppm): 163.6(C-11′,13′), 159.9(C-11,13,11″,13″), 158.8 (C-4,4″), 158.7 (C-4′), 148.2 (C-9,9″), 135.1 (C-9′), 134.1 (C-1, 1″), 132.3 (C-7′), 131.1 (C-1′), 129.5 (C-2′, 6′), 128.6 (C-2,6,2″,6″), 124.6 (C-8′), 121.2 (C-10′,14′), 116.8 (C-3, 5, 3″,5″), 116.6 (C-3′, 5′), 107.6 (C-10, 14,10″,14″), 102.4 (C-12, 12″), 95.6 (C-7, 7″), 92.3 (C-12′), 59.2 (C-8, 8″).

#### *trans*-Suffruticosol D (9)

UV λ_max_ nm: 203, 230 (sh), 282 (MeOH). ESI–MS *m/z*: 679 (M^+^–H). ^1^H-NMR (500 MHz, CD_3_OD) δ (ppm): 7.19 (2H, d, *J *= 8.5 Hz, H- 2, 6), 6.98 (2H, d, *J* = 8.5 Hz, H-2″, 6″), 6.79 (2H, d, *J *= 8.5 Hz, H-3, 5), 6.75 (2H, d, *J* = 8.5 Hz, H-2′, 6′), 6.56 (2H, d, *J* = 8.5 Hz, H-3″, 5″), 6.54 (2H, d, *J *= 8.5 Hz, H-3′, 5′), 6.51 (H, d, *J *= 16.0 Hz, H-7′), 6.46 (H, s, H-12′), 6.43 (H, d, *J* = 16.0 Hz, H-8′), 6.19 (H, t, *J *= 2.0 Hz, H-12), 6.17 (2H, d, *J *= 2.0 Hz, H-10,14), 5.94 (H, t, *J* = 2.0 Hz, H-12″), 5.83 (H, d, *J* = 8.0 Hz, H-7″), 5.78 (2H, d, *J *= 2.0 Hz, H-10″,14″), 5.43 (H, d, *J *= 6.0 Hz, H-7), 4.52 (H, d, *J *= 8.0 Hz, H-8″), 4.39 (H, d, *J *= 6.0 Hz, H-8). ^13^C-NMR (125 MHz, CD_3_OD) δ (ppm):163.4 (C-13′), 162.8 (C-11′), 160.7 (C-11, 13), 159.5 (C-11″,13″), 159.1 (C-4), 158.9 (C-4′), 158.2 (C-4″), 148.1 (C-9), 144.1 (C- 9″), 134.9 (C-7′), 134.6 (C-1), 134.1 (C-9′), 131.1 (C-1′), 130.0 (C-1″), 129.8 (C-2″,6″), 129.1 (C-2′, 6′), 128.6 (C-2, 6), 122.9 (C-8′), 122.6 (C-14′), 121.7 (C-10′), 116.9 (C-3, 5), 116.8 (C-3″,5″), 115.8 (C-3′, 5′), 109.7 (C-10″,14″), 107.9 (C-10, 14), 102.7 (C-12), 102.3 (C-12″), 95.4 (C-7), 92.4 (C-12′), 91.9 (C-7″), 59.3 (C-8), 55.3 (C-8″).

#### *trans*-gnetin H (10)

UV λ_max_ nm: 230, 325 (MeOH). ESI–MS *m/z*: 679 (M^+^ - H). ^1^H-NMR (500 MHz, CD_3_OD) δ (ppm): 7.18 [4H, dd, *J *= 2.0, 8.5 Hz, H-2(6), 2″ (6″)], 6.78 [4H, dd, *J *= 2.0, 8.5 Hz, H-3(5), 3″ (5″)], 6.69 (2H, d, *J *= 8.5 Hz, H-2′, 6′), 6.51 (2H, d, *J *= 8.5 Hz, H-3′, 5′), 6.42 (1H, s, *J *= 8.5 Hz, H-12), 6.38 (2H, s, H-7′, 8′), 6.14 [6H, s, H-10(10″), 12(12″), 14(14″)], 5.40 (2H, d, *J *= 5.5 Hz, H-7, 7″), 4.40 (2H, d, *J *= 5.5 Hz, H-8, 8″). ^13^C-NMR (125 MHz, CD_3_OD) δ (ppm):163.5 (C-11′, 13′), 160.6 [C-11(11″), 13(13″)], 159.0 (C-4, 4″), 158.8 (C-4′), 148.0 (C-9, 9″), 135.0 (C-9′), 134.7 (C-1″), 134.2 (C-1), 131.1 (C-1′), 129.2 (C-2′, 6′, 8′), 128.6 [C-2(2″), 6(6″)], 123.0 (C-7′), 120.8 (C-10′, 14′), 116.9 [C-3(3″), 5(5″)], 116.7 (C-3′, 5′), 107.8 [C-10(10″), 14(14″)], 102.7 (C-12, 12″), 95.3 (C-7, 7″), 92.0 (C-12′), 59.4 (C-8, 8″).

The purity of ten compounds were detected using the protocol previously described by He et al. [31]. Briefly, an Agilent 1200 HPLC system (Agilent Technologies, Palo Alto, CA, USA) equipped with an online vacuum degasser, a quaternary pump, an autosampler, a thermostated column compartment, and a diode array detector was used. HPLC separation was performed by a YMC-pack ODS-A column (250 mm × 4.6 mm, 5 μm) using the mobile phase containing water (A) and methanol (B) in a gradient: 0–10 min, linear gradient from 35% to 52% B; 10–30 min, linear gradient from 52% to 60% B. The flow rate was at 1.0 mL/min. The column temperature was set at 25 °C. The injection volume was 5 μL, and the UV detection wavelength was set at 230 nm. The retention times of **1** to **10** were 9.23 min, 10.61 min, 11.18 min, 13.56 min, 15.39 min, 18.80 min, 20.31 min, 21.63 min, 25.68 min and 26.51 min, respectively. The purities of suffruticosol A, suffruticosol B, suffruticosol C, *trans*-resveratrol, *cis*-*ε*-viniferin, *trans*-*ε*-viniferin, *cis*-suffruticosol D, *cis*-gnetin H, *trans*-suffruticosol D, and *trans*-gnetin H were determined by HPLC using normalization of the peak area and were 98.2%, 96.1%, 98.6%, 98.6%, 95.8%, 98.1%, 95.5%, 94.5%, 86.7%, and 85.8%, respectively (shown in Additional file 5).

Finally, the compounds were resuspended in dimethyl sulfoxide (DMSO) to yield a concentration of 50 mM and were stored at 4 °C.

## Rabbit articular cartilage culture

The primary articular chondrocytes from knee joints of young adult New Zealand white rabbits were provided by iCell Bioscience Inc. We further used Collagen II immunofluorescence to confirm the purity of chondrocytes was over 90% (shown in Additional file 6), which guaranteed the credibility of the results. Cells were cultured at a density of 10^5^ cells/well in DMEM/HG medium supplemented with 10% fetal bovine serum (FBS), 100 × penicillin and streptomycin/mL at 37 °C, 5% CO_2_, 95% humidity. We choose the third generation of chondrocytes for experiments, and cells were cultured with 0.5% FBS for 12 h to be starved. Except for the normal control group, 10 ng/mL IL-1β would be added to the cultures for 24 h to make the OA chondrocyte model.

We explored relevant conditions. As a result, we treated the OA chondrocytes with 1 μM diacerein as a positive control to compare the efficiency with oligostilbenes. The seed extract of *Paeonia suffruticosa* or oligostilbenes were added in each group of OA chondrocytes separately for 24 h to investigate their influence. The DMSO content in each group was adjusted to 0.1%.

### Cell viability assay

Chondrocytes were seeded in 96-well plates at 10,000 cells per well in three replicates. After treating each group with drug for 24 h as above, the medium was replaced with 100 μL of CCK8 premix (10 μL CCK8 + 90 μL DMEM/HG medium). In addition, then plates were incubated for 2 h in and incubator kept at 5% CO_2_, 37 °C. The data were assayed by microplate reader (Bio-Rad, USA) at 450 nm. Cell viability was calculated as a percentage of the treated group relative to the normal control group.

### Analysis of apoptosis using Annexin V sand PI staining

After treating cells by oligostilbenes for 24 h as above, the adherent cells were collected by 0.25% trypsin without EDTA. Then, they were centrifuged at 2000 rpm for 5–10 min and washed with PBS. Then, 500 μL Binding Buffer (100 mM HEPES, 140 mM NaCl, 25 mM CaCl_2_, pH 7.4) were added to suspended cells. Finally, 5 μL Annexin V-FITC and PI labeled antibodies were added to each sample for 15 min in the dark. All operations were conducted at room temperature (25 °C). Flow cytometry data were plotted and analyzed by the fluorescence-activated cell-sorting system (Accuri C6 Plus, Bio-Rad, USA) within 1 h of staining.

### Real-time PCR analysis

Total RNA was obtained from lysates of rabbit chondrocytes by the standard TRIzol method and was then reverse *trans*cribed into cDNA. Primers (shown in Table 2) were synthesized by Ige Biotech Company (Guangzhou, China) and were diluted to 2.5 μM with RNase-free water. The reaction mix (10 μL of SYBR Green Supermix, 0.8 μL of PCR Forward Primer, 0.8 μL of PCR Reverse Primer and 3.4 μL of dH_2_O) was prepared on ice and then was added to a PCR tube containing 5 μL of cDNA template and was centrifuged at 100 g. The reaction procedure was as follows: 1. predenaturation 95 °C 30 s, 1 cycle; 2. 95 °C 5 s, 55 °C 30 s, 72 °C 1 min, 40 cycles; 3. 95 °C 15 s, 60 °C 30 s, 95 °C 15 s, END. The data were analyzed by the ΔΔCT method. All RT reactions were performed in triplicate.

**Table 2:** Primer sequences used in real-time PCR

Gene	Forward primer	Reverse primer
GAPDH	5′-ATCCATTCATTGACCTCCACTAC-3′	5′-GTACTGGGCACCAGCATCAC-3′
SOX9	5′-GGGAAGCTCTGGAGACTGCT-3′	5′-TGTAGTCCGGGTGGTCTTTC -3′
COL2A1	5′-AGAAGAACTGGTGGAGCAGCAAGA-3′	5′-TTTACAAGAAGCAGACGGGCCCTA-3′
MMP13	5′-TTGACCACTCCAAGGACCCAG-3′	5′-GAGGATGCAGACGCCAGAAGA-3′

### Statistical analysis

The data analysis was performed by GraphPad Prism6 (GraphPad Software, USA) and Excel (Microsoft, USA). The two-tailed, unpaired t test was used for differential analysis between the two groups. P-values < 0.05 were considered to be statistically significant differences. The results are presented as the mean values ± standard error (SE) with 95% confidence intervals (CI).

## Additional files

**Additional file 1.** Morphological changes of OA chondrocytes under the action of different concentrations of *Paeonia suffruticosa* seed extract. Chondrocytes were treated with 10 ng/mL IL-1β and with different concentrations (0, 0.01, 0.1, 0.5, 1, 5 and 10 mg/L) of seed extract for 24 h. Untreated chondrocytes as control group. Panel A indicates the state of cells just before treatment. Images showed that their morphology and quantity were similar. Panel B indicates the state of cells after treatment. Compared with control group, IL-1β group obviously decreased in cell number, with irregular shapes and disordered arrangement. After adding low concentration (0.01–1 μM) of extract, the number of cells increased relatively and their morphology were closer to normal chondrocytes. But the apoptotic cells increased significantly after treatment with higher concentration of extract (10 μM), which indicated the cytotoxicity of extract. Scale bar = 200 μm.

**Additional file 2.** Morphological changes of OA chondrocytes under the action of 0.1 mg/L *Paeonia suffruticosa* seed extract and ten oligostilbenes. Cells were treated with 10 ng/mL IL-1β and 0.1 mg/L of a different drug for 24 h. Untreated chondrocytes as control group. (1) suffruticosol A, (2) suffruticosol B, (3) suffruticosol C, (4) *trans*-resveratrol, (5) *cis*-ε-viniferin, (6) *trans*-ε-viniferin, (7) *cis*-suffruticosol D, (8) *cis*-gnetin H, (9) *trans*-suffruticosol D, and (10) *trans*-gnetin H. Images showed that 0.1 mg/L *Paeonia suffruticosa* seed extract and ten oligostilbenes could promote proliferation of OA chondrocytes in some degree and made the cell morphology closer to normal chondrocytes. Scale bar = 200 μm.

**Additional file 3.** Flowchart of the extraction and isolation of oligostilbenes from *Paeonia suffruticosa* seed. 1 suffruticosol A, 2 suffruticosol B, 3 suffruticosol C, 4 *trans*-resveratrol, 5 *cis*-ε-viniferin, 6 *trans*-ε-viniferin, 7 *cis*-suffruticosol D, 8 *cis*-gnetin H, 9 *trans*-suffruticosol D, and 10 *trans*-gnetin H.

**Additional file 4.** Spectrograms of structure identification of ten compounds. ^1^H- and ^13^C-NMR spectra of ten compounds were measured on a Bruker Avance DRX-500 spectrometer (^1^H at 500 MHz and ^13^C at 125 MHz) in MeOH-*d*4. Chemical shifts are given in δ values (ppm) relative to tetramethylsilane (TMS) as an internal standard. 1 suffruticosol A, 2 suffruticosol B, 3 suffruticosol C, 4 *trans*-resveratrol, 5 *cis*-ε-viniferin, 6 *trans*-ε-viniferin, 7 *cis*-suffruticosol D, 8 *cis*-gnetin H, 9 *trans*-suffruticosol D, and 10 *trans*-gnetin H.

**Additional file 5.** Chromatograms of *P. suffruticosa* seed extract and ten oligostilbenes for purity detection. (1) suffruticosol A, (2) suffruticosol B, (3) suffruticosol C, (4) *trans*-resveratrol, (5) *cis*-ε-viniferin, (6) *trans*-ε-viniferin, (7) *cis*-suffruticosol D, (8) *cis*-gnetin H, (9) *trans*-suffruticosol D, and (10) *trans*-gnetin H. HPLC separation was performed by a YMC-pack ODS-A column (250 mm × 4.6 mm, 5 μm) using the mobile phase containing water (A) and methanol (B) in a gradient. The flow rate was at 1.0 mL/min and the UV detection wavelength was set at 230 nm. The retention times of 1 to 10 (Peak numbers correspond to numbers in Table 1) were 9.23 min, 10.61 min, 11.18 min, 13.56 min, 15.39 min, 18.80 min, 20.31 min, 21.63 min, 25.68 min and 26.51 min, respectively. The purities of them were determined by HPLC using normalization of the peak area and were 98.2%, 96.1%, 98.6%, 98.6%, 95.8%, 98.1%, 95.5%, 94.5%, 86.7%, and 85.8%, respectively.

**Additional file 6.** Immunofluorescence of Collagen II to identify primary chondrocytes. The Collagen Type II Antibody (Proteintech, USA) was used as the primary antibody. After the chondrocytes were incubated with goat anti-mouse immunoglobulin Alexa Fluor Plus 594 (1:200 dilution), they were stained red. The nuclei were stained with DAPI. After merging the images, it could be seen that the purity of chondrocytes was over 90%.

## Data Availability

The datasets and samples of the compounds are available from the authors.

## Abbreviations

CCK-8: Cell Counting Kit 8
DMSO: dimethyl sulfoxide
EC_50_: concentration for 50% of maximal effect
ECM: extracellular matrix
EtOH: ethyl alcohol
FBS: fetal bovine serum
HPLC: High Performance Liquid Chromatography
NSAIDS: nonsteroidal anti-inflammatory drugs
OA: osteoarthritis
TMS: tetramethylsilane
