# Supplementary figures and images for: Protective effects of ten oligostilbenes from Paeonia suffruticosa seeds on interleukin-1β-induced rabbit osteoarthritis chondrocytes
Source: BMC Chem. 2019 May 23;13(1):72. doi: 10.1186/s13065-019-0589-4 (PMC6661769; doi:10.1186/s13065-019-0589-4)

**A**

Control

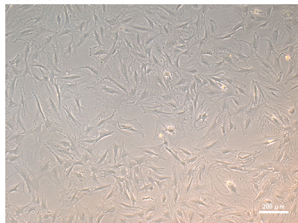IL-1 $\beta$ 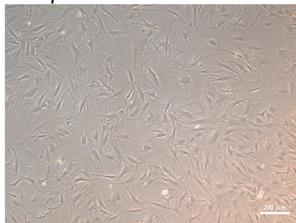

0.01

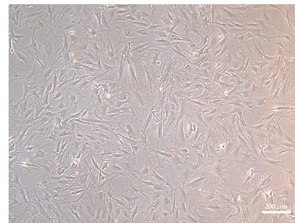

0.1

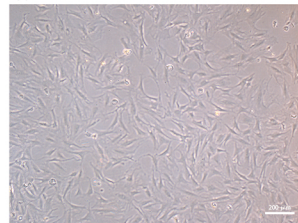

0.5

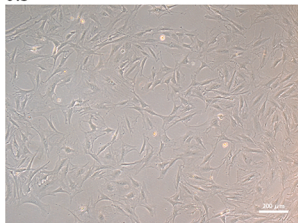

1

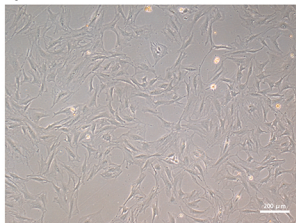

5

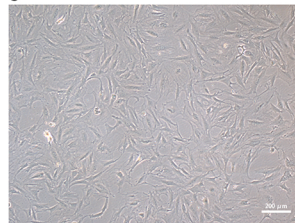

10

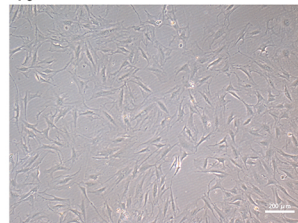**B**

Control

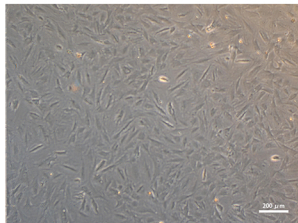IL-1 $\beta$ 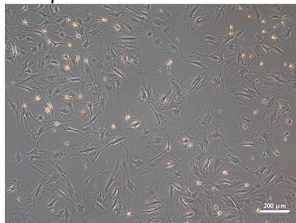

0.01

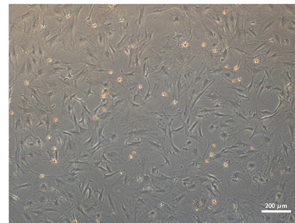

0.1

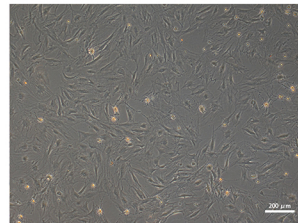

0.5

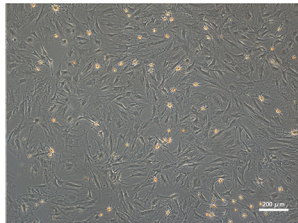

1

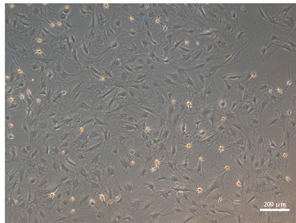

5

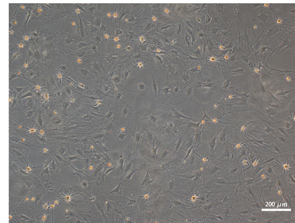

10

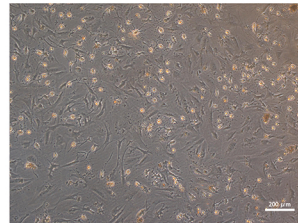

Supplement: Supplementary file 1 — Additional file 1. Morphological changes of OA chondrocytes under the action of different concentrations of Paeonia suffruticosa seed extract. Chondrocytes were treated with 10 ng/mL IL-1β and with different concentrations (0, 0.01, 0.1, 0.5, 1, 5 and 10 mg/L) of seed extract for 24 h. Untreated chondrocytes as control group. Panel A indicates the state of cells just before treatment. Images showed that their morphology and quantity were similar. Panel B indicates the state of cells after treatment. Compared with control group, IL-1β group obviously decreased in cell number, with irregular shapes and disordered arrangement. After adding low concentration (0.01–1 μM) of extract, the number of cells increased relatively and their morphology were closer to normal chondrocytes. But the apoptotic cells increased significantly after treatment with higher concentration of extract (10 μM), which indicated the cytotoxicity of extract. Scale bar = 200 μm. [file 13065_2019_589_MOESM1_ESM.pdf]

Control

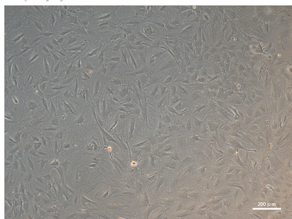

IL-1  $\beta$

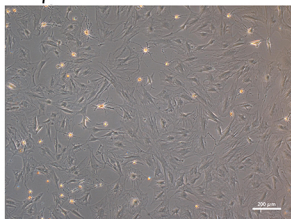

Diacerein

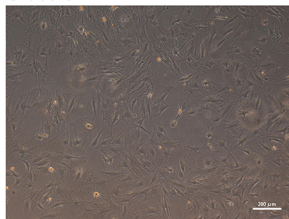

Extract

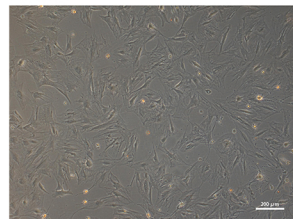

1

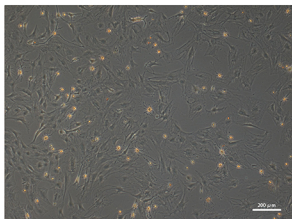

2

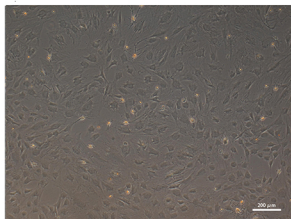

3

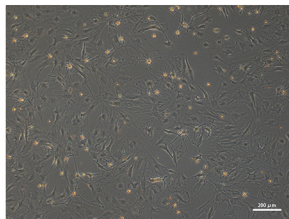

4

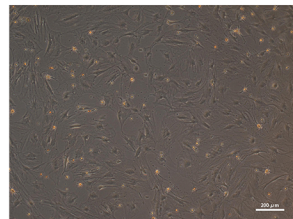

5

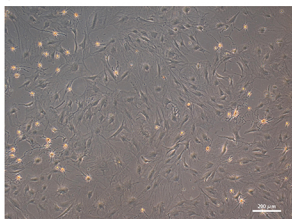

6

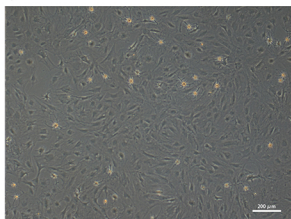

7

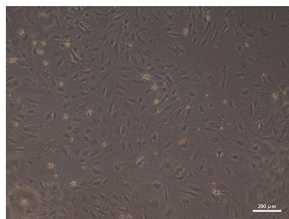

8

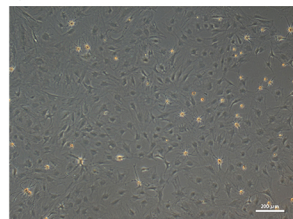

9

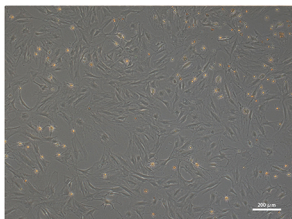

10

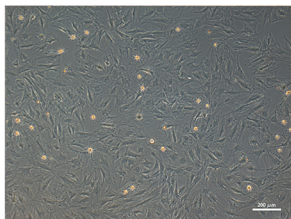

Supplement: Supplementary file 2 — Additional file 2. Morphological changes of OA chondrocytes under the action of 0.1 mg/L Paeonia suffruticosa seed extract and ten oligostilbenes. Cells were treated with 10 ng/mL IL-1β and 0.1 mg/L of a different drug for 24 h. Untreated chondrocytes as control group. (1) suffruticosol A, (2) suffruticosol B, (3) suffruticosol C, (4) trans-resveratrol, (5) cis-ε-viniferin, (6) trans-ε-viniferin, (7) cis-suffruticosol D, (8) cis-gnetin H, (9) trans-suffruticosol D, and (10) trans-gnetin H. Images showed that 0.1 mg/L Paeonia suffruticosa seed extract and ten oligostilbenes could promote proliferation of OA chondrocytes in some degree and made the cell morphology closer to normal chondrocytes. Scale bar = 200 μm. [file 13065_2019_589_MOESM2_ESM.pdf]

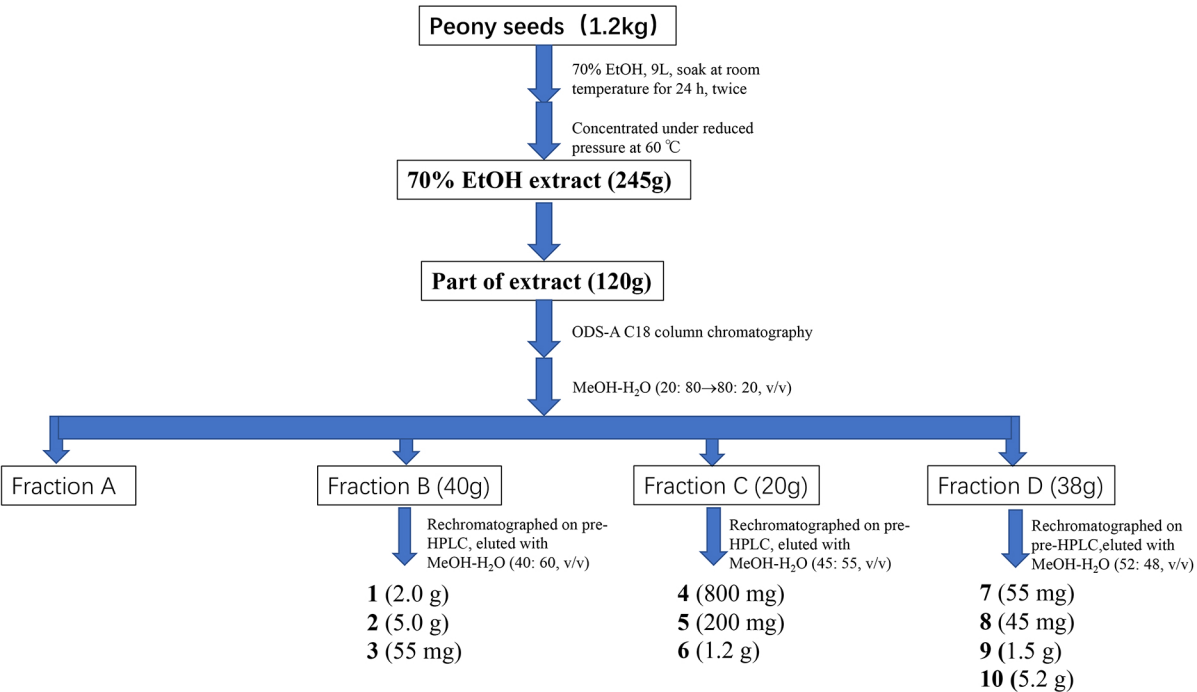

Supplement: Supplementary file 3 — Additional file 3. Flowchart of the extraction and isolation of oligostilbenes from Paeonia suffruticosa seed. 1 suffruticosol A, 2 suffruticosol B, 3 suffruticosol C, 4 trans-resveratrol, 5 cis-ε-viniferin, 6 trans-ε-viniferin, 7 cis-suffruticosol D, 8 cis-gnetin H, 9 trans-suffruticosol D, and 10 trans-gnetin H. [file 13065_2019_589_MOESM3_ESM.pdf]

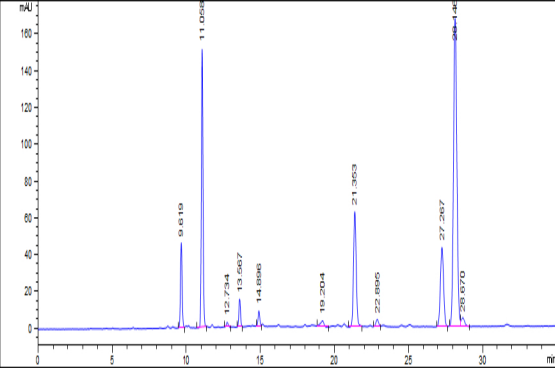

Extract

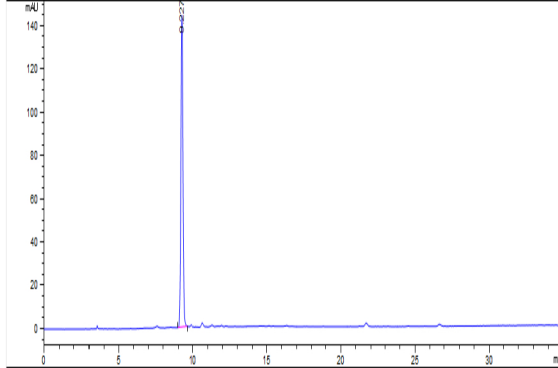

(1)

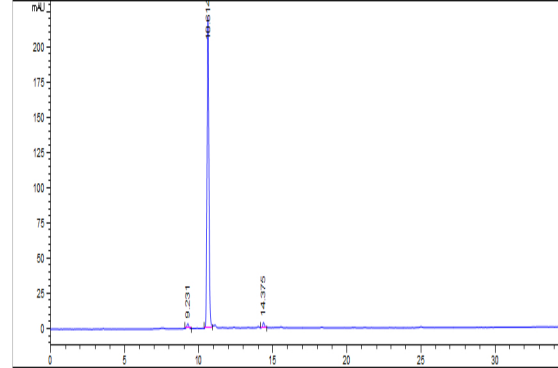

(2)

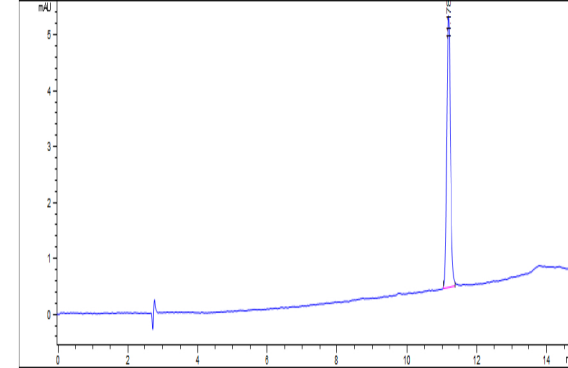

(3)

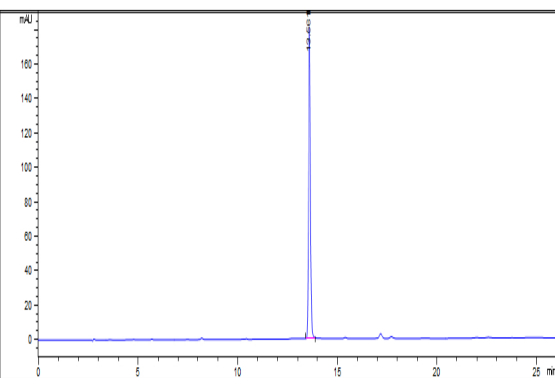

(4)

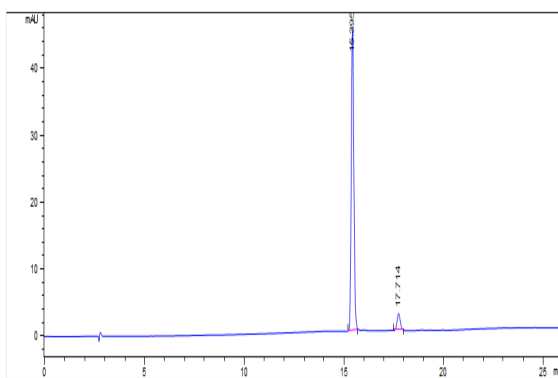

(5)

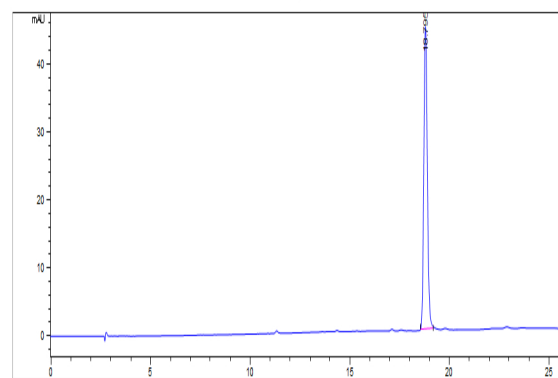

(6)

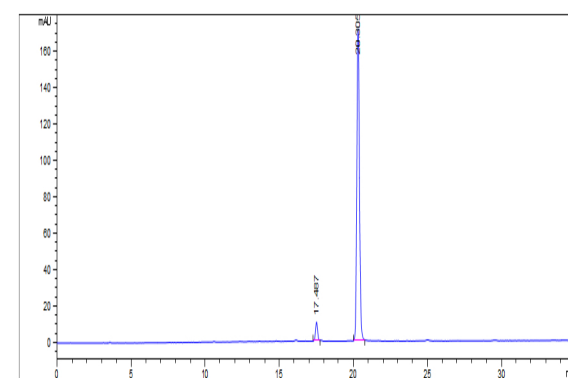

(7)

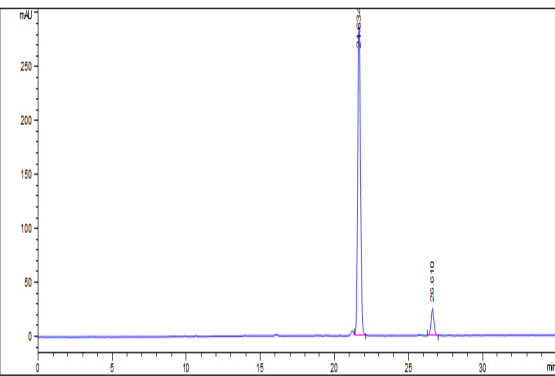

(8)

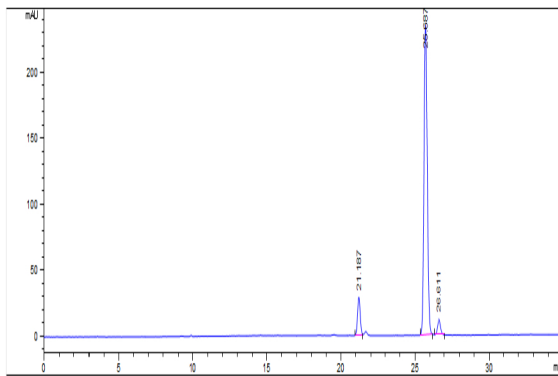

(9)

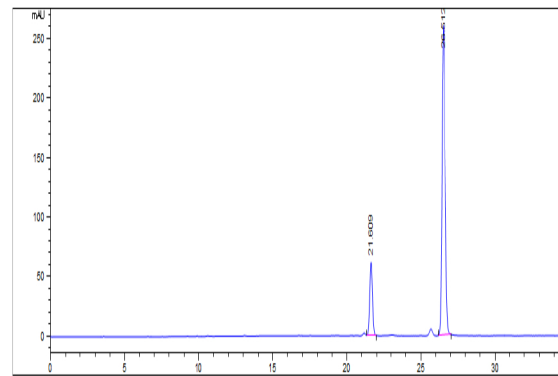

(10)

Supplement: Supplementary file 5 — Additional file 5. Chromatograms of P. suffruticosa seed extract and ten oligostilbenes for purity detection. (1) suffruticosol A, (2) suffruticosol B, (3) suffruticosol C, (4) trans-resveratrol, (5) cis-ε-viniferin, (6) trans-ε-viniferin, (7) cis-suffruticosol D, (8) cis-gnetin H, (9) trans-suffruticosol D, and (10) trans-gnetin H. HPLC separation was performed by a YMC-pack ODS-A column (250 mm × 4.6 mm, 5 μm) using the mobile phase containing water (A) and methanol (B) in a gradient. The flow rate was at 1.0 mL/min and the UV detection wavelength was set at 230 nm. The retention times of 1 to 10 (Peak numbers correspond to numbers in Table 1) were 9.23 min, 10.61 min, 11.18 min, 13.56 min, 15.39 min, 18.80 min, 20.31 min, 21.63 min, 25.68 min and 26.51 min, respectively. The purities of them were determined by HPLC using normalization of the peak area and were 98.2%, 96.1%, 98.6%, 98.6%, 95.8%, 98.1%, 95.5%, 94.5%, 86.7%, and 85.8%, respectively. [file 13065_2019_589_MOESM5_ESM.pdf]

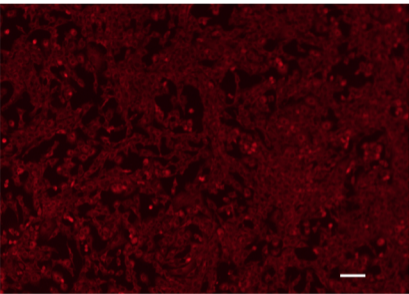

100X-F

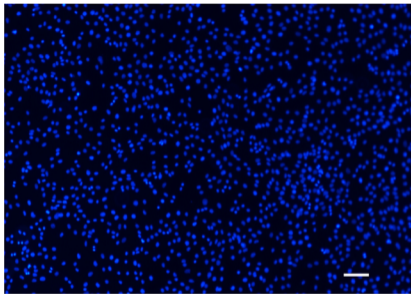

100X-DAPI

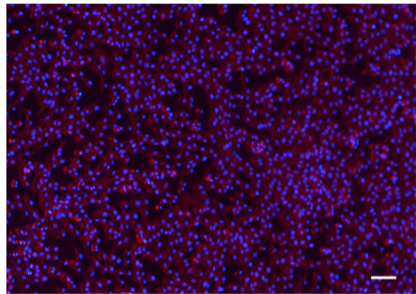

Merge

$\text{— } 100\text{ }\mu\text{m}$

Supplement: Supplementary file 6 — Additional file 6. Immunofluorescence of Collagen II to identify primary chondrocytes. The Collagen Type II Antibody (Proteintech, USA) was used as the primary antibody. After the chondrocytes were incubated with goat anti-mouse immunoglobulin Alexa Fluor Plus 594 (1:200 dilution), they were stained red. The nuclei were stained with DAPI. After merging the images, it could be seen that the purity of chondrocytes was over 90%. [file 13065_2019_589_MOESM6_ESM.pdf]
