# Supplementary material for: Protective effects of ten oligostilbenes from Paeonia suffruticosa seeds on interleukin-1β-induced rabbit osteoarthritis chondrocytes
Source: BMC Chem. 2019 May 23;13(1):72. doi: 10.1186/s13065-019-0589-4 (PMC6661769; doi:10.1186/s13065-019-0589-4)
Supplement: Supplementary file 4 — Additional file 4. Spectrograms of structure identification of ten compounds. 1H- and 13C-NMR spectra of ten compounds were measured on a Bruker Avance DRX-500 spectrometer (1H at 500 MHz and 13C at 125 MHz) in MeOH-d4. Chemical shifts are given in δ values (ppm) relative to tetramethylsilane (TMS) as an internal standard. 1 suffruticosol A, 2 suffruticosol B, 3 suffruticosol C, 4 trans-resveratrol, 5 cis-ε-viniferin, 6 trans-ε-viniferin, 7 cis-suffruticosol D, 8 cis-gnetin H, 9 trans-suffruticosol D, and 10 trans-gnetin H. [file 13065_2019_589_MOESM4_ESM.pdf]

1

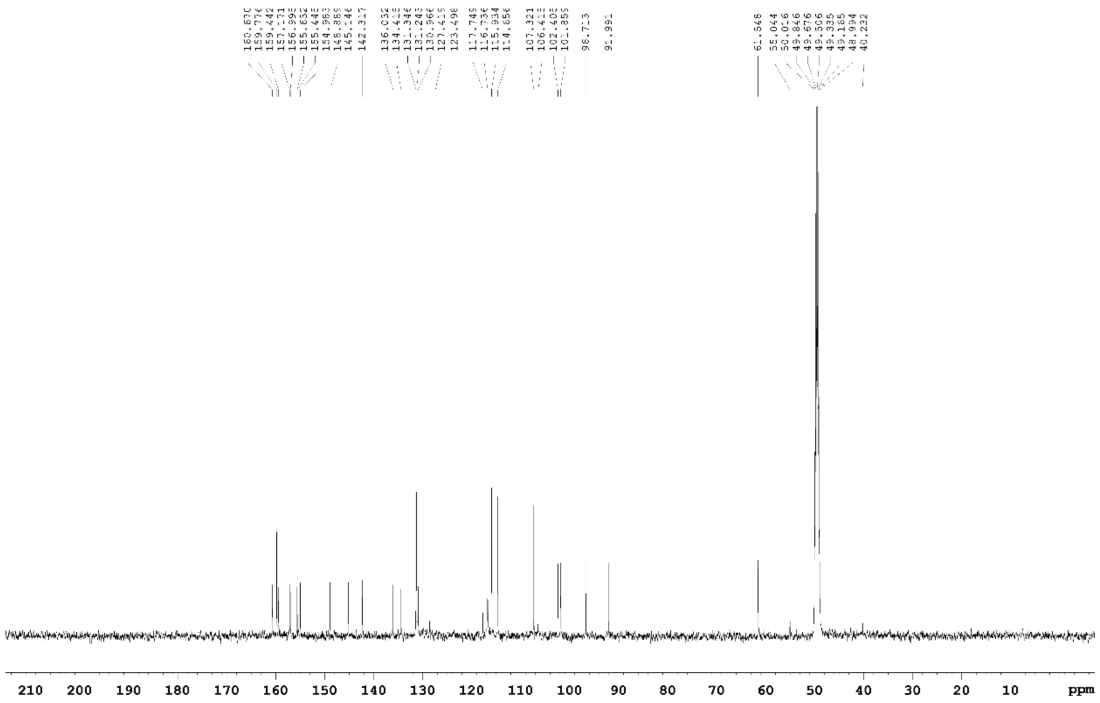

^{13}C-NMR (125 MHz, CD_3OD) of **Suffruticosol A**

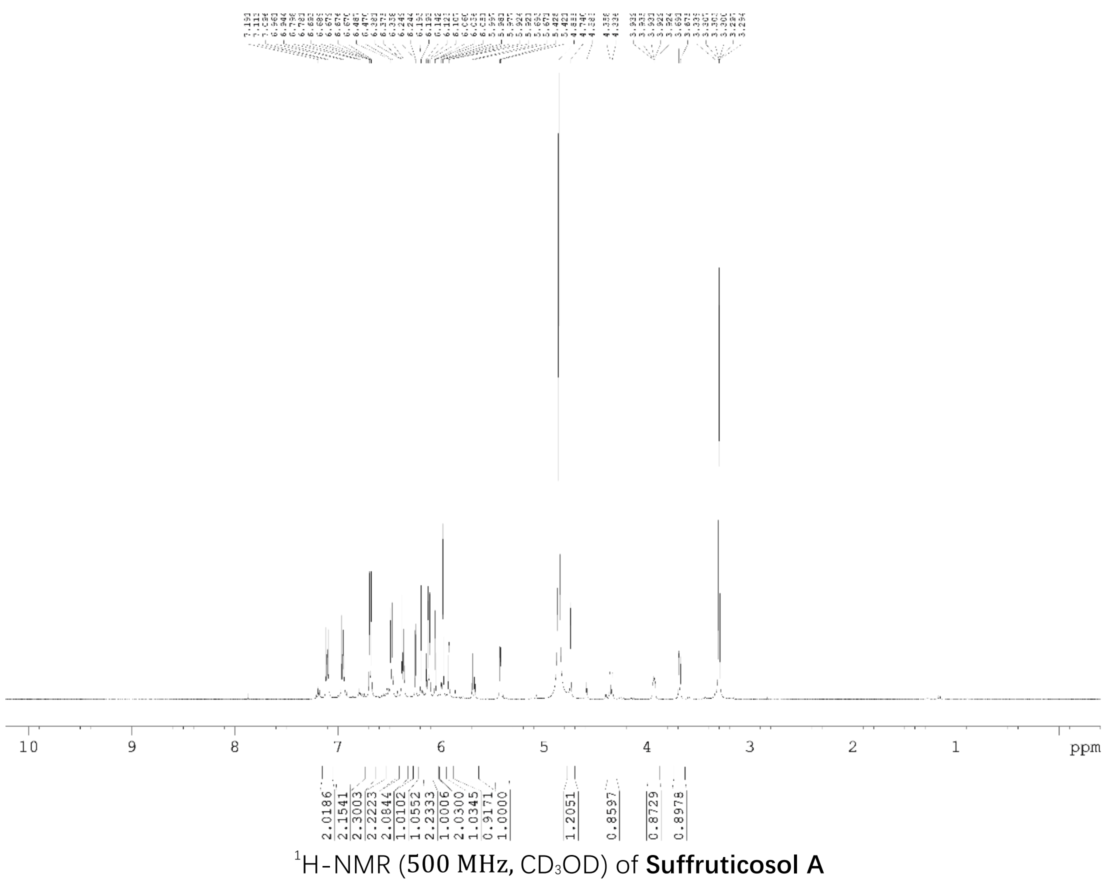

^{1}H-NMR (500 MHz, CD_3OD) of **Suffruticosol A**

2

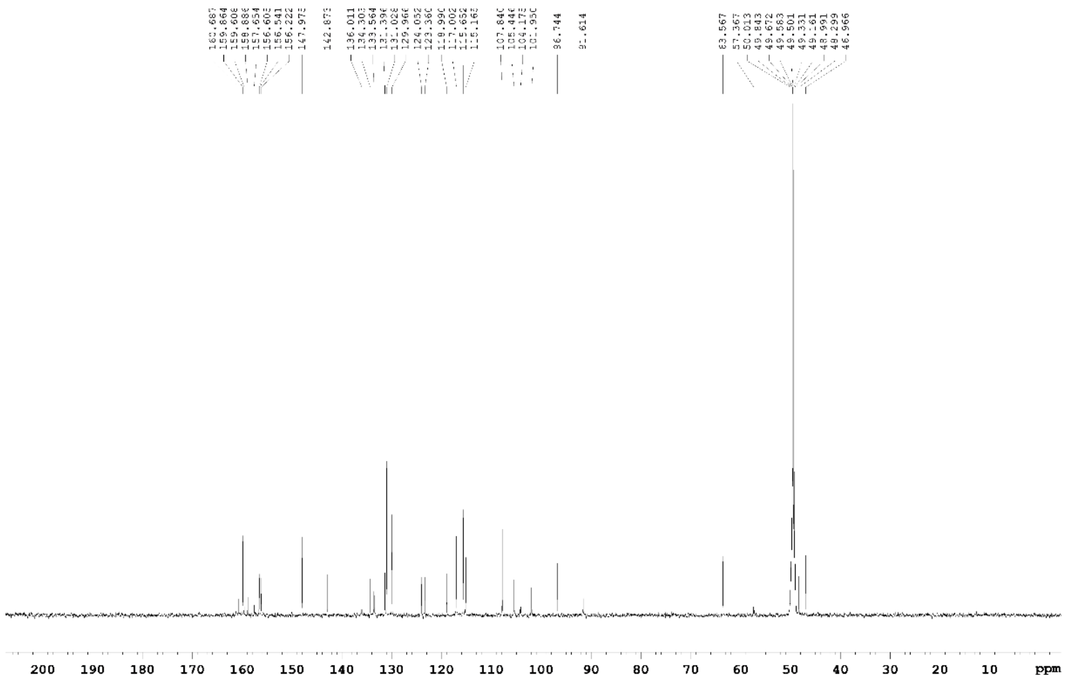

^{13}C-NMR (125 MHz, CD_3OD) of **Suffruticosol B**

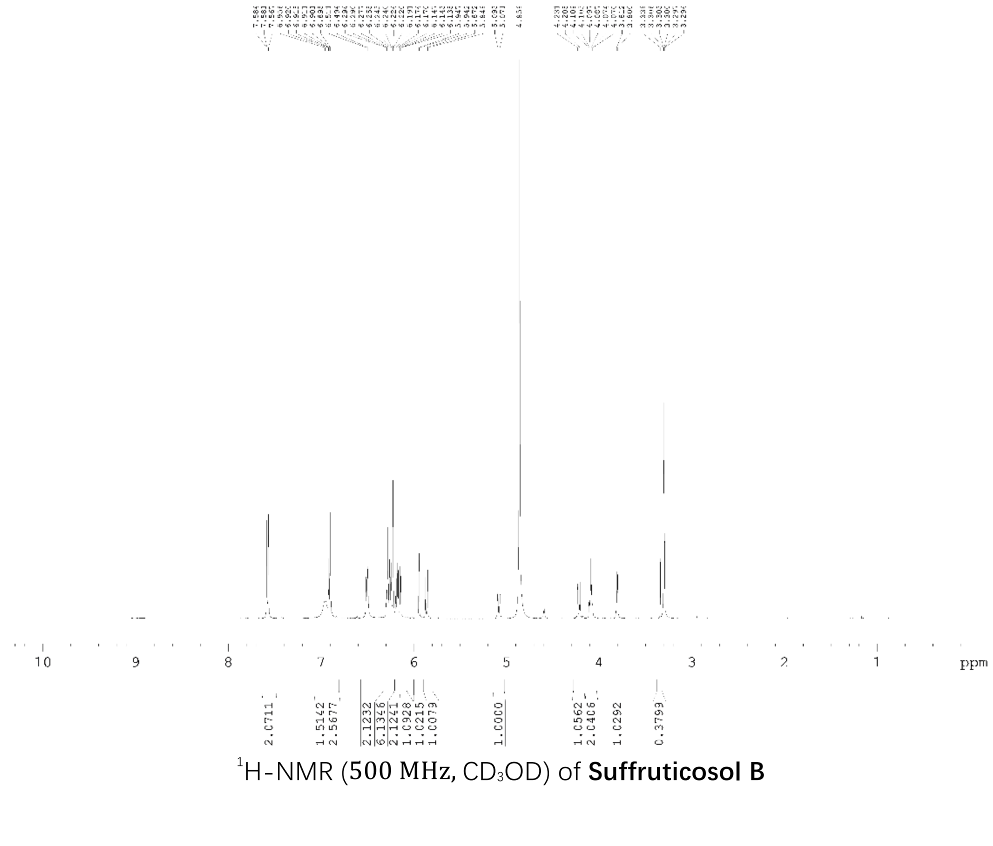

^{1}H-NMR (500 MHz, CD_3OD) of **Suffruticosol B**

3

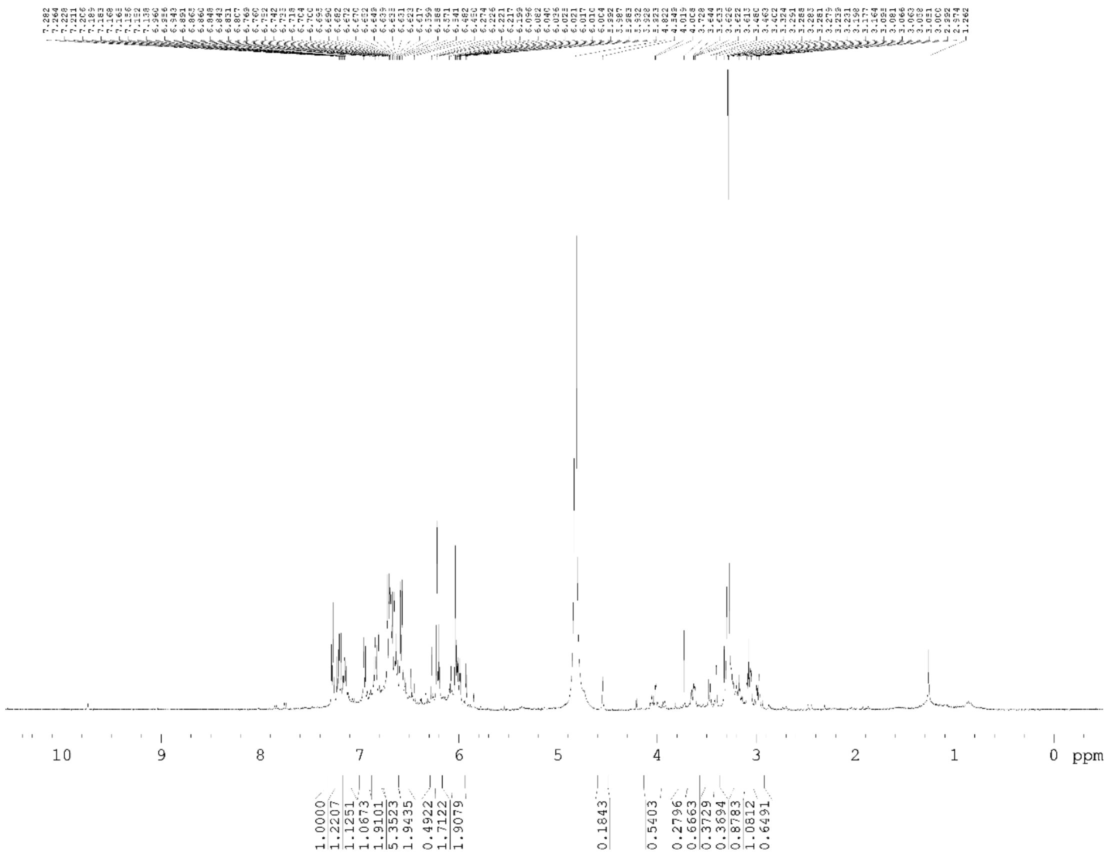

^{1}H-NMR (500 MHz, CD_3OD) of **Suffruticosol C**

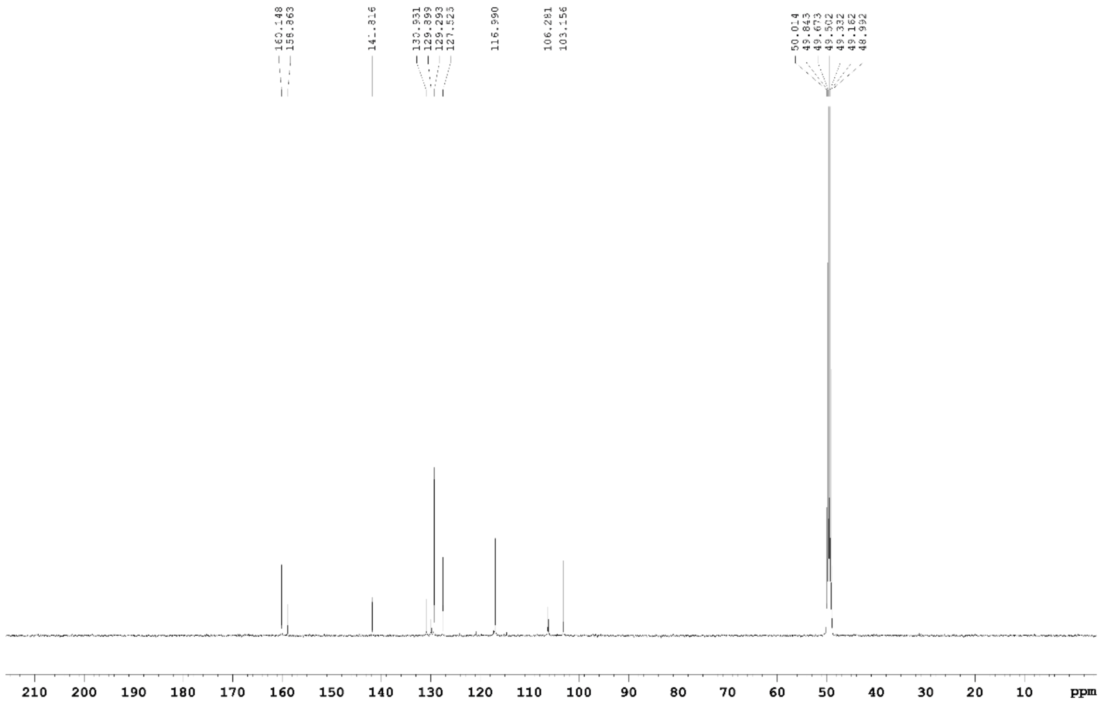

^{13}C-NMR (125 MHz, CD_3OD) of **Resveratrol**

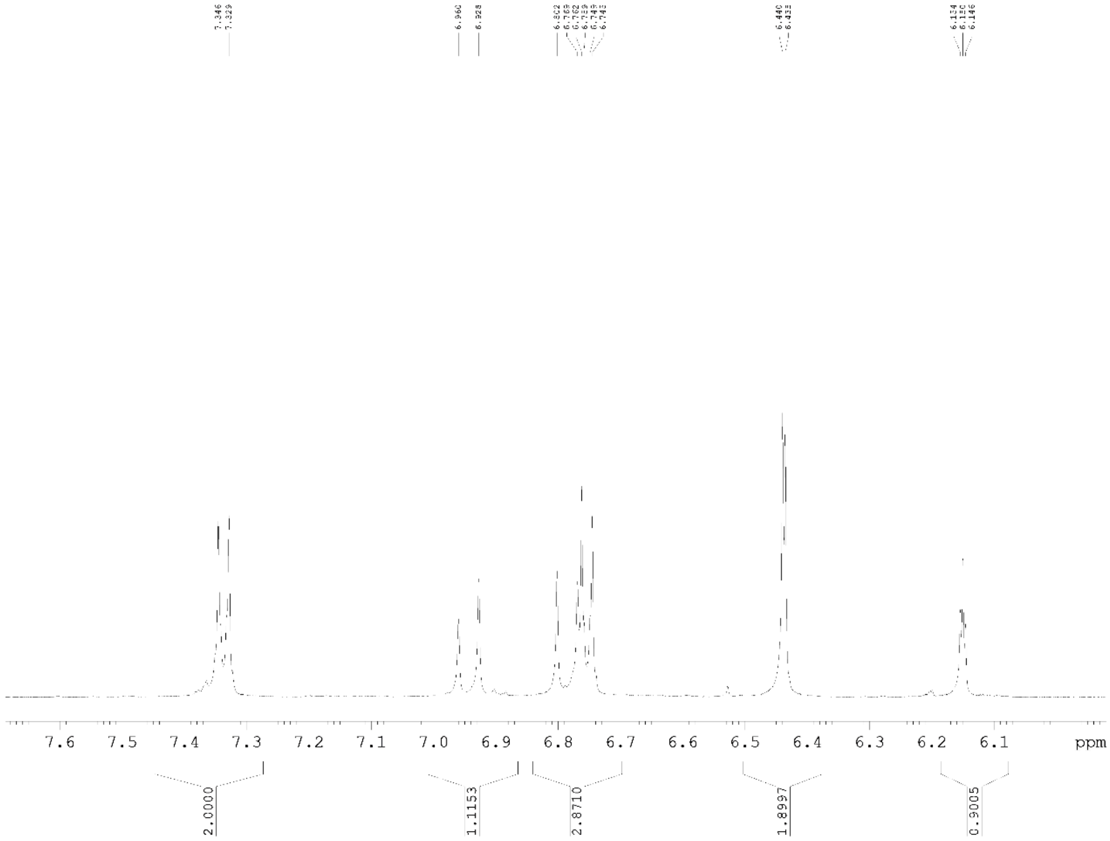

^{1}H-NMR (500 MHz, CD_3OD) of **Resveratrol**

5

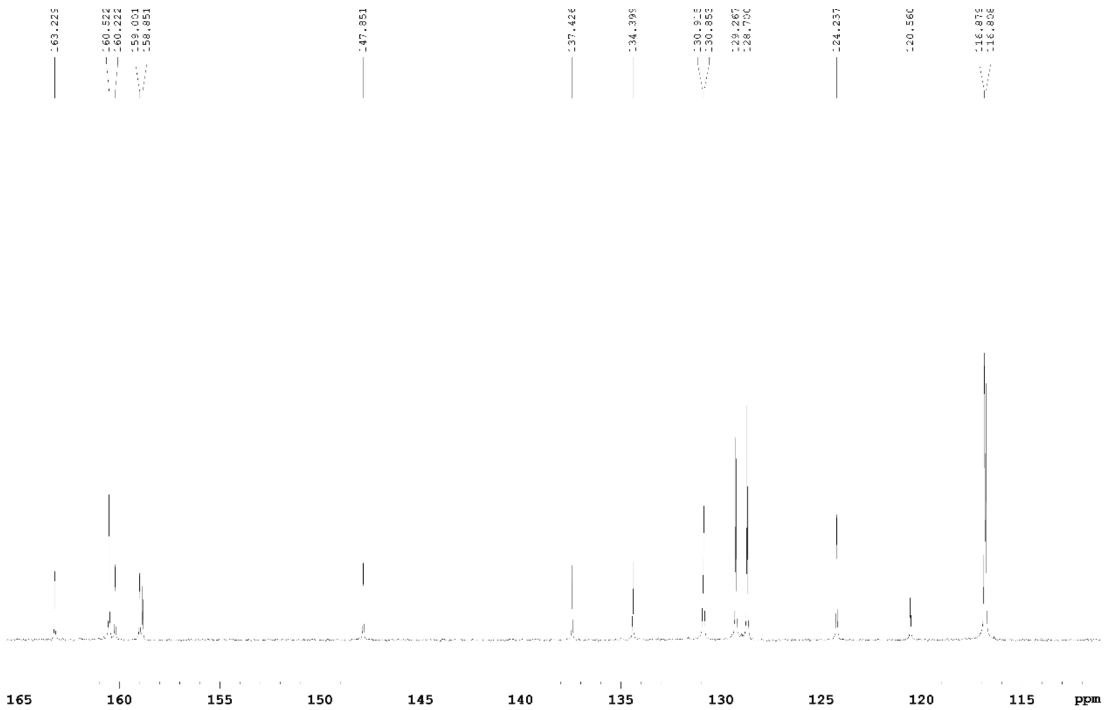

^{13}C-NMR (125 MHz, CD_3OD) of ***cis*-*ε*-Viniferin**

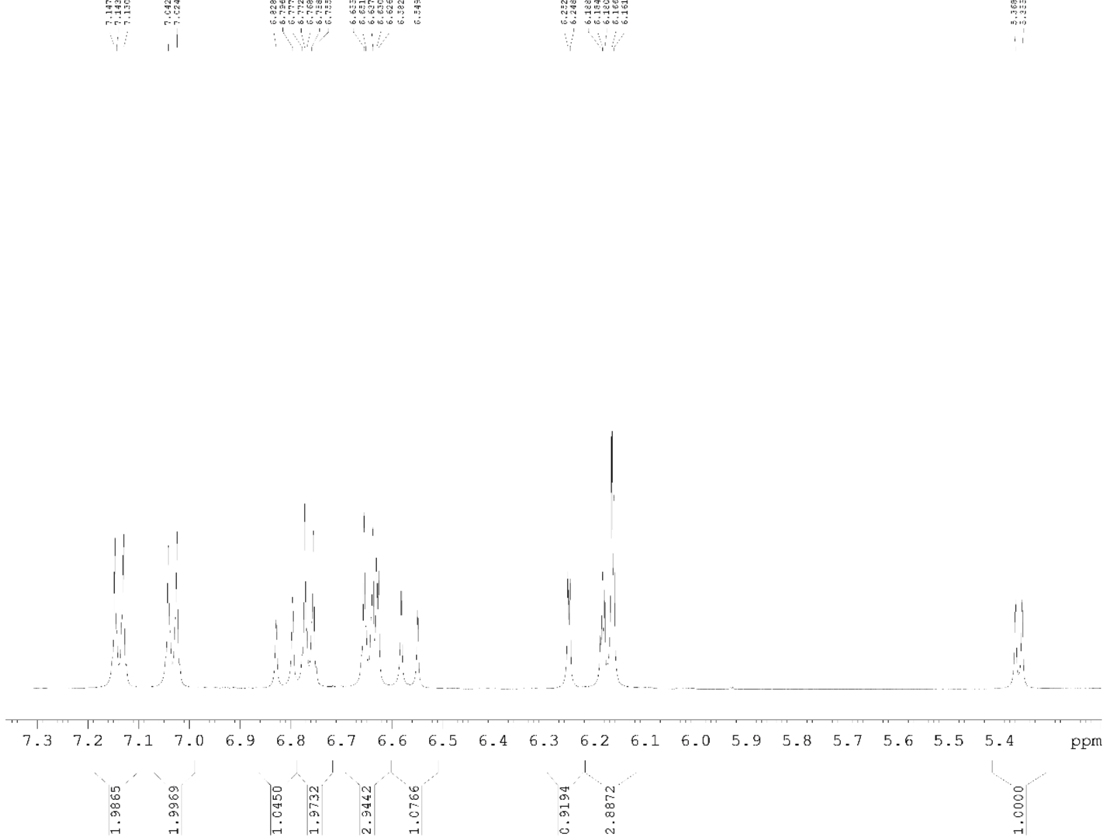

^{1}H-NMR (500 MHz, CD_3OD) of ***cis*-*ε*-Viniferin**

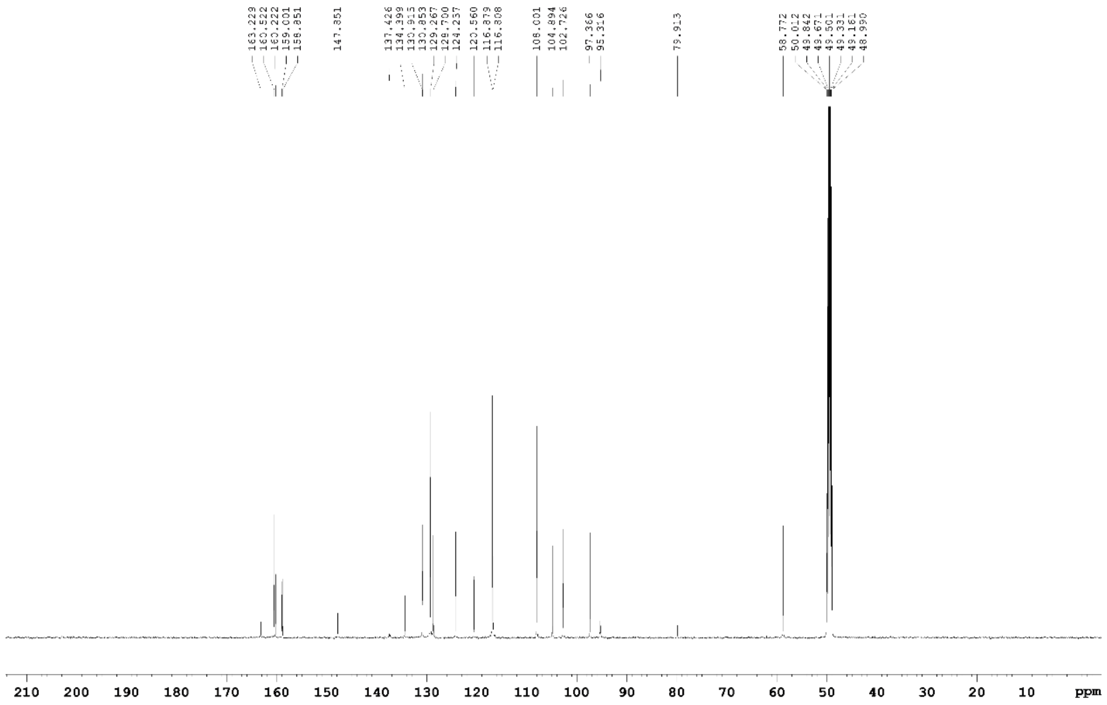

^{13}C-NMR (125 MHz, CD_3OD) of ***trans-ε*-Viniferin**

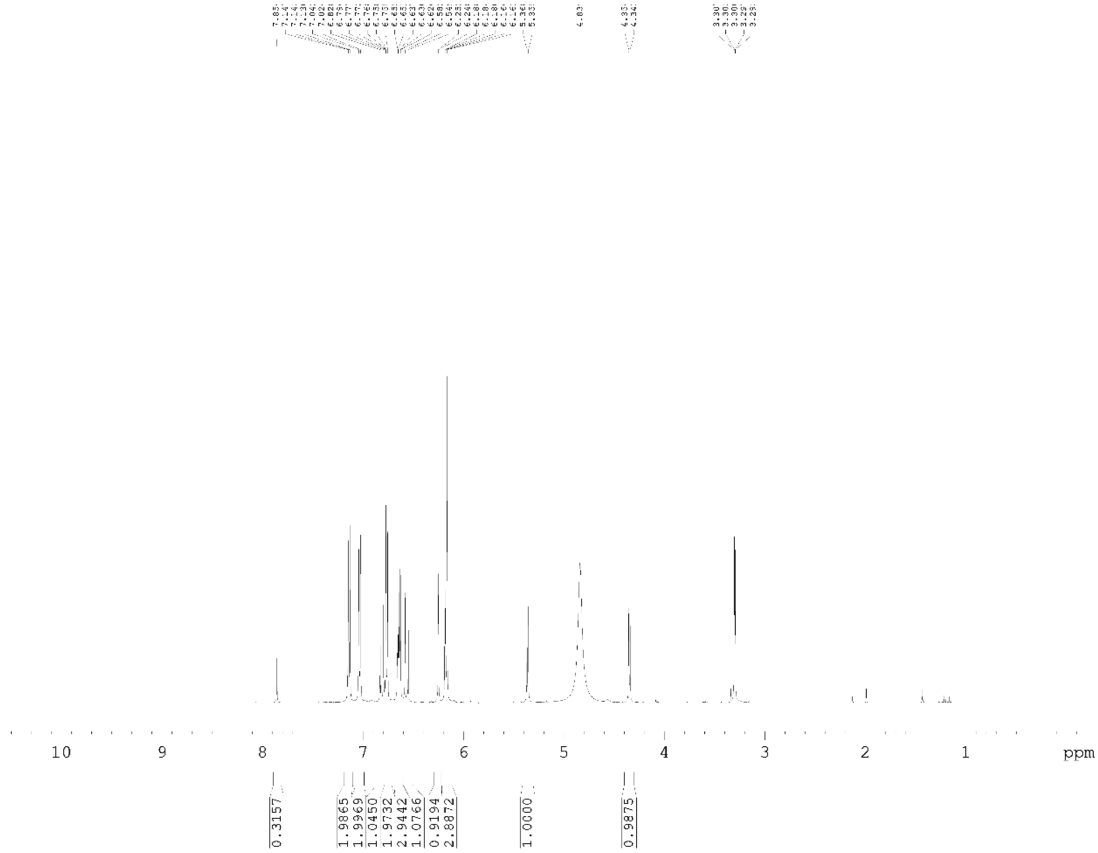

^{1}H-NMR (500 MHz, CD_3OD) of ***trans-ε*-Viniferin**

7

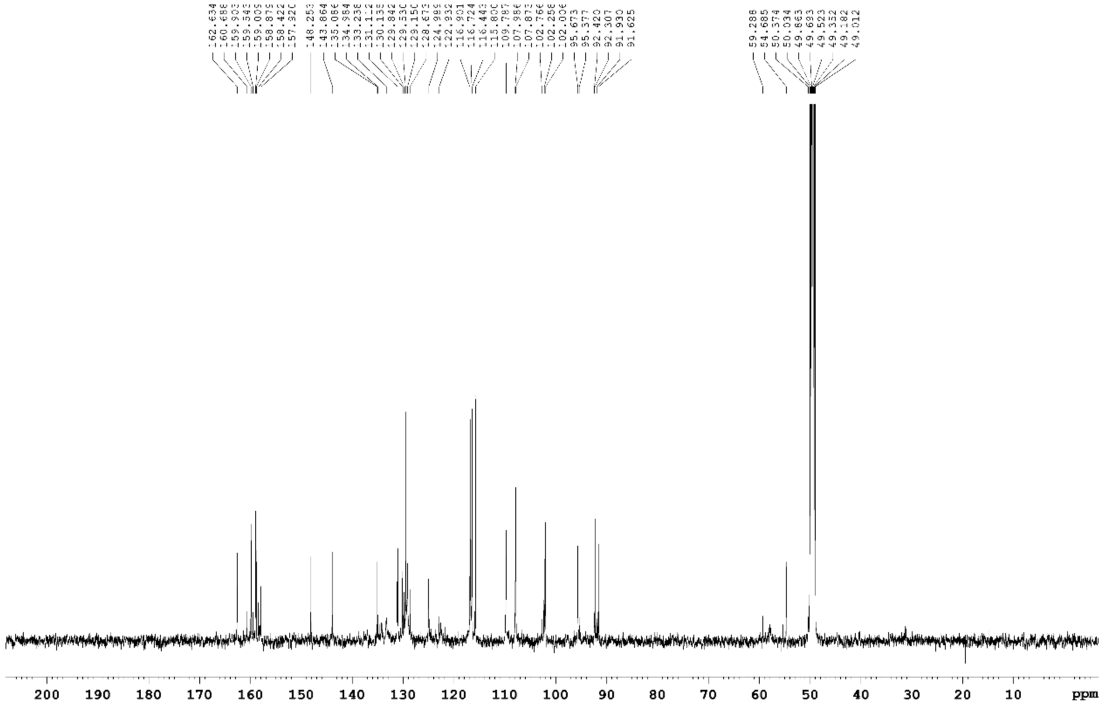

^{13}C-NMR (125 MHz, CD_3OD) of ***cis*-Suffruticosol D**

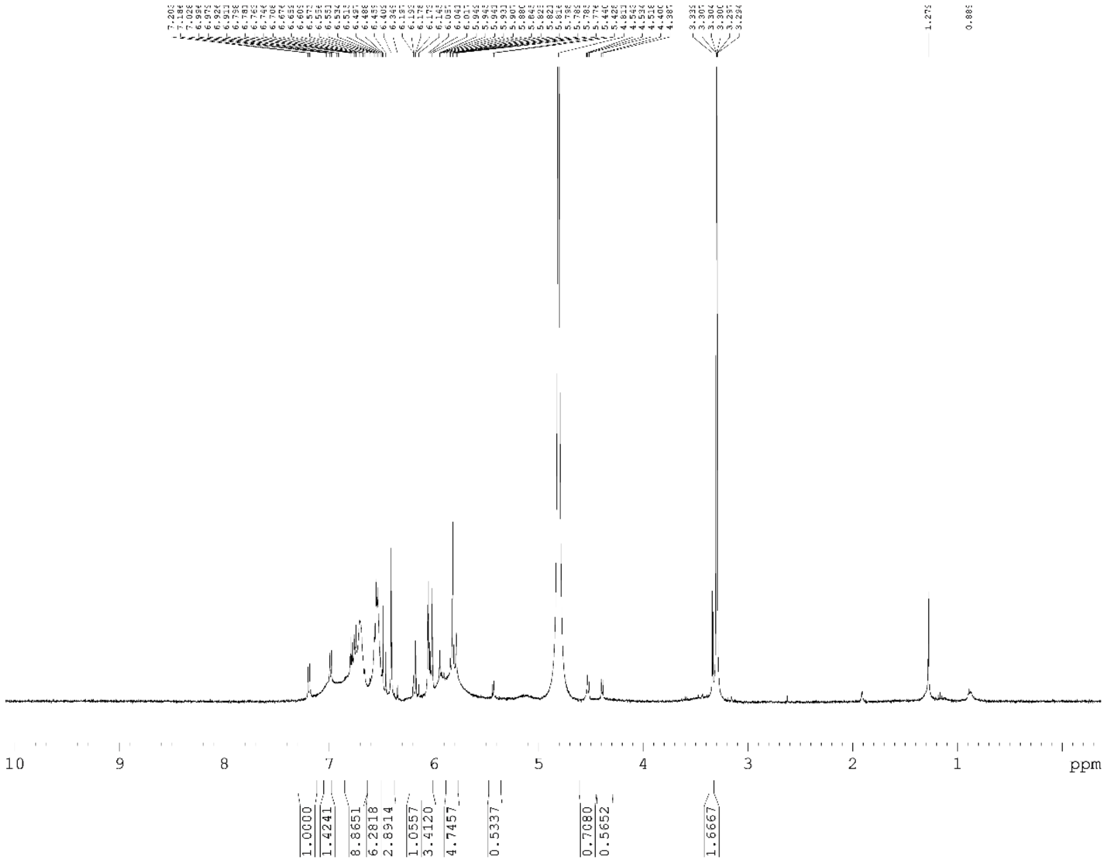

^{1}H-NMR (500 MHz, CD_3OD) of ***cis*-Suffruticosol D**

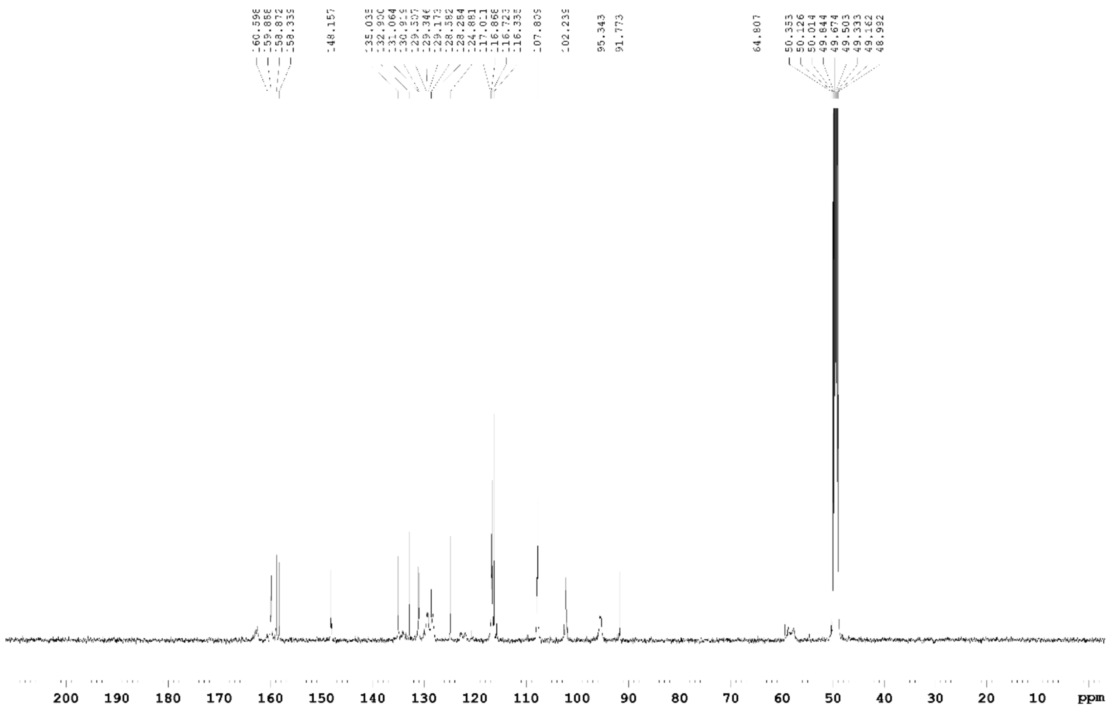

^{13}C-NMR (125 MHz, CD_3OD) of ***cis*-gnetin H**

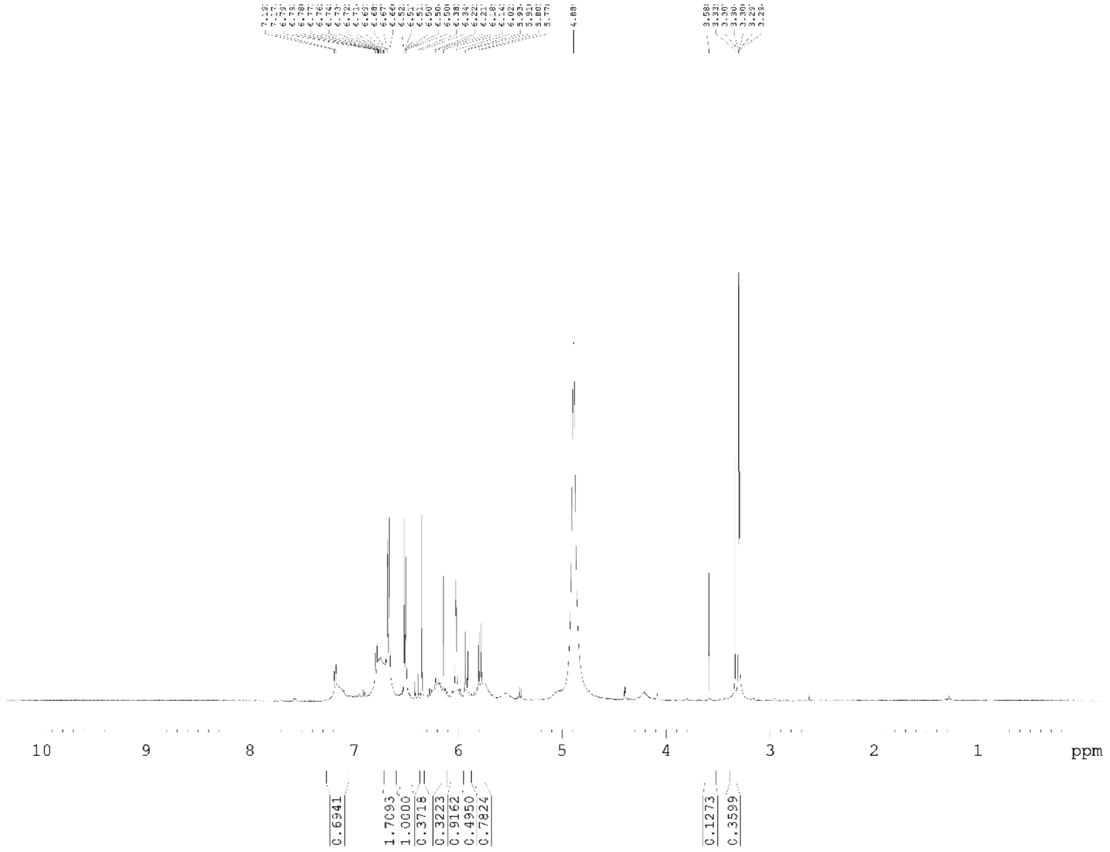

^{1}H-NMR (500 MHz, CD_3OD) of ***cis*-gnetin H**

9

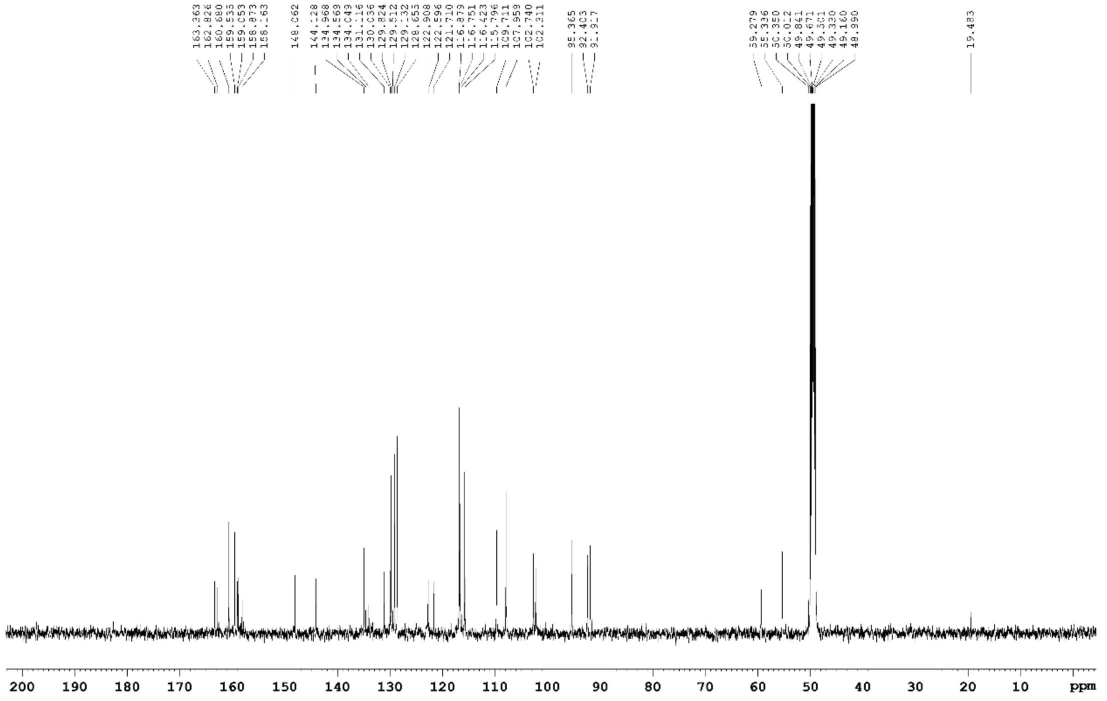

^{13}C-NMR (125 MHz, CD_3OD) of ***trans*-Suffruticosol D**

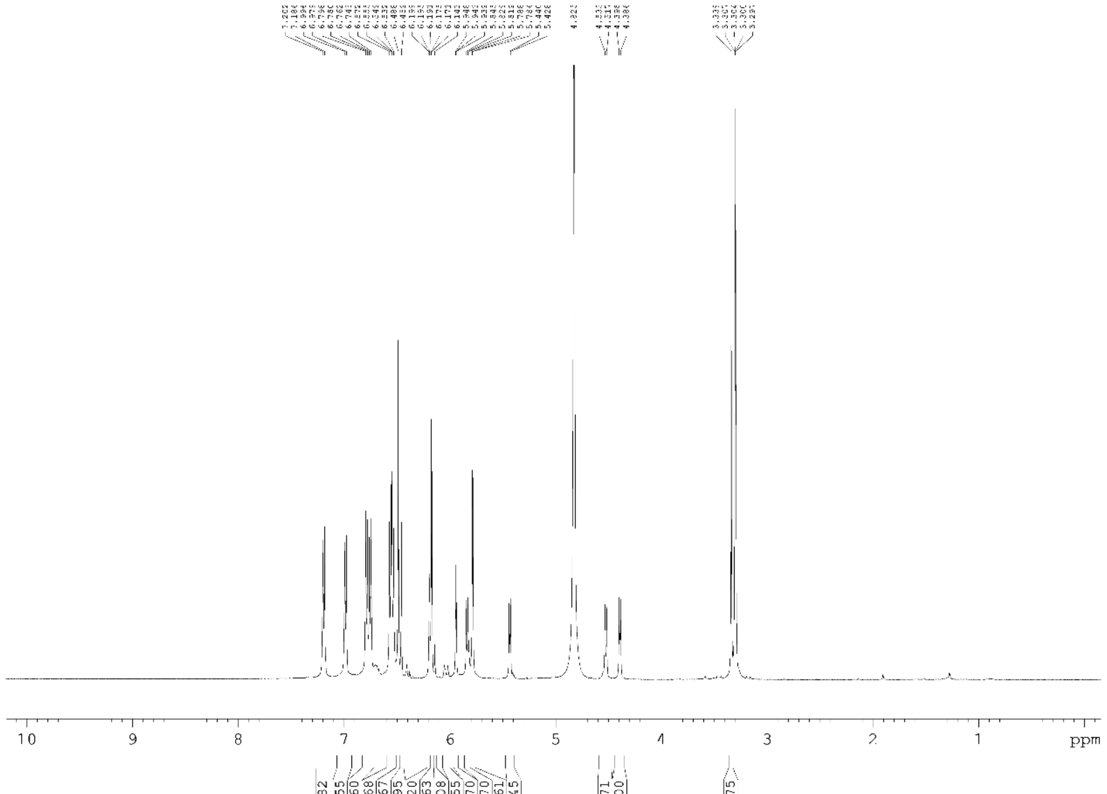

^{1}H-NMR (500 MHz, CD_3OD) of ***trans*-Suffruticosol D**

10

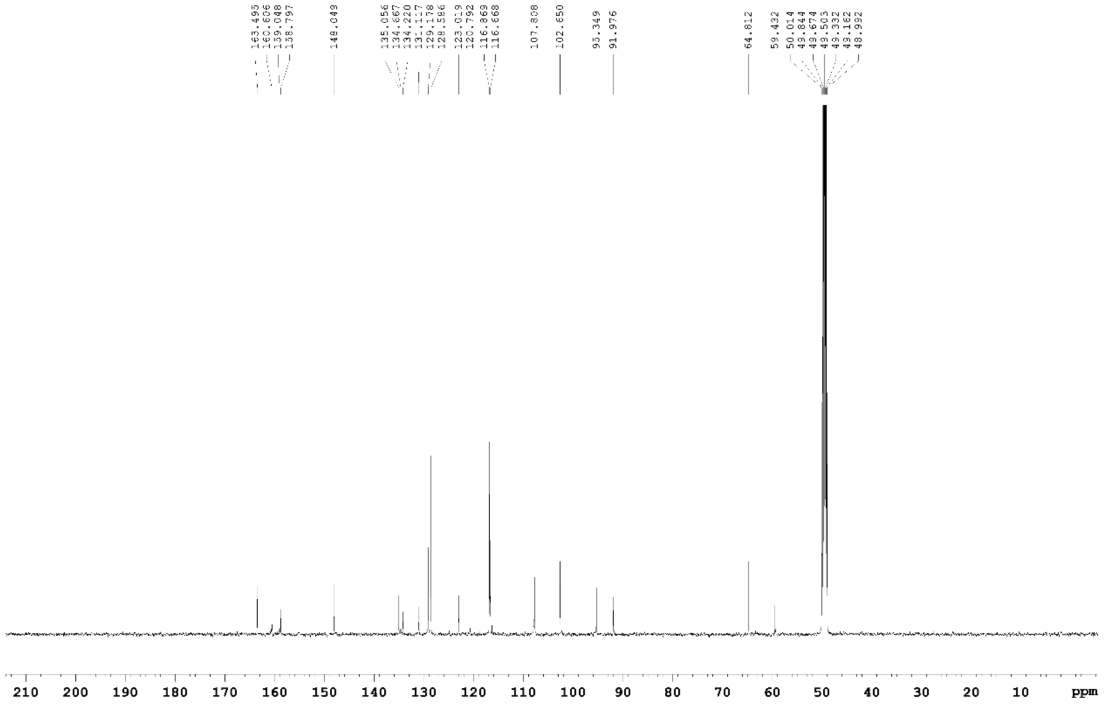

^{13}C-NMR (125 MHz, CD_3OD) of ***trans*-gnetin H**

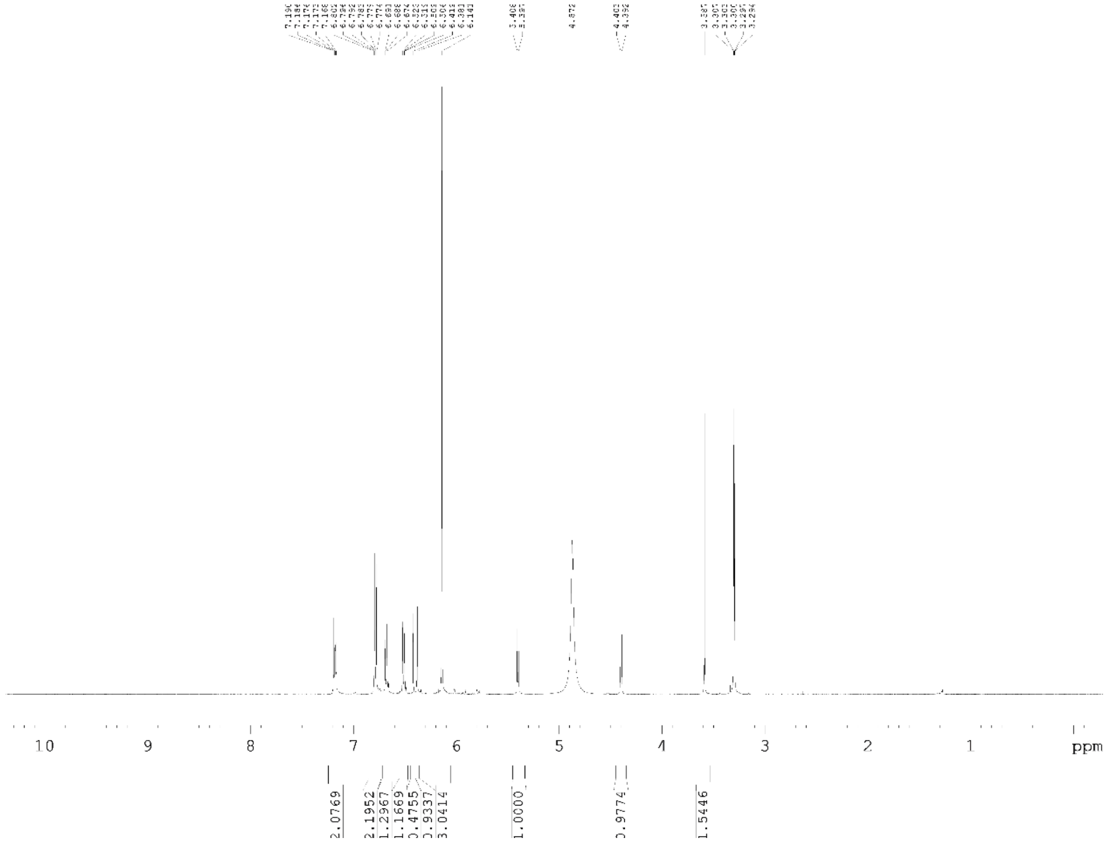

^{1}H-NMR (500 MHz, CD_3OD) of ***trans*-gnetin H**
